# Predicting In‐Hospital Fall Risk Using Machine Learning With Real‐Time Location System and Electronic Medical Records

**DOI:** 10.1002/jcsm.13713

**Published:** 2025-02-24

**Authors:** Dong Won Kim, Jihoon Seo, Sujin Kwon, Chan Min Park, Changho Han, Yujeong Kim, Jaewoong Kim, Chul Sik Kim, Seok Won Park, Dukyong Yoon, Kyoung Min Kim

**Affiliations:** ^1^ Department of Biomedical Systems Informatics Yonsei University College of Medicine Seoul Republic of Korea; ^2^ Department of Endocrinology, Internal Medicine, Yongin Severance Hospital Yonsei University College of Medicine Yongin Republic of Korea; ^3^ Institute for Innovation in Digital Healthcare Severance Hospital Seoul Republic of Korea; ^4^ Center for Digital Health, Yongin Severance Hospital Yonsei University Health System Yongin Republic of Korea

**Keywords:** in‐hospital fall risk prediction, machine learning, real‐time location system

## Abstract

**Background:**

Hospital falls are the most prevalent and fatal event in healthcare, posing significant risks to patient health outcomes and institutional care quality. Real‐time location system (RTLS) enables continuous tracking of patient location, providing a unique opportunity to monitor changes in physical activity, a key factor related to the risk of falls in hospitals. This study is aimed at utilizing RTLS data to capture dynamic patient movements, integrating it with clinical information through a machine learning approach to enhance in‐hospital fall predictions.

**Methods:**

This retrospective study developed and compared three models: clinical data only, RTLS data only and a combined data model. It included 22 201 patients from Yongin Severance Hospital, South Korea, from March 2020 to June 2022, with 118 fall patients and 443 nonfall patients selected through random sampling and relevant criteria for detailed analysis, totaling 561 patients. The average age of the participants was 70.1 years, with a median of 71.0 years (IQR: 60.0–80.0). Among participants, 52.6% (*n* = 295) were male. This study evaluated the occurrence of the first fall during hospitalization. The performance was assessed using the area under the receiver operating characteristic (AUROC), the area under the precision‐recall curve (AUPRC) and the Brier score. The Shapley additive explanations (SHAP) method and decision curve analysis (DCA) were employed to enhance model explainability and assess the clinical utility of the models.

**Results:**

The RTLS model showed significant predictive accuracy for hospital falls, with an AUROC of 0.813 (95% CI: 0.703–0.903). The clinical + RTLS model outperformed those using only one type of data, achieving an AUROC of 0.847 (95% CI: 0.764–0.917), AUPRC of 0.667 (95% CI: 0.472–0.816) and Brier score of 0.120 (95% CI: 0.083–0.162), with significant differences in performance metrics (*p* < 0.0001). DCA confirmed its greater clinical benefit. SHAP analysis indicated that patients who experienced falls tended to have less active time and slower movement speed just before the fall compared to the early hospitalization period, despite attempting to move more. Additionally, higher fall incidence was significantly associated with sedative use and higher red cell distribution width (RDW) levels.

**Conclusion:**

This study underscores the capability of utilizing RTLS to predict in‐hospital falls by tracking the changes of patients' physical activity through a machine learning approach. This may improve early fall risk detection during hospitalization, thereby preventing falls and enhancing patient safety.

## 1 Introduction

Managing patient safety and hospital operations is crucial in the healthcare domain. In‐hospital falls are among the most common incidents that cause concern in healthcare facilities. This issue not only affects patient health outcomes but also has substantial economic implications. For instance, in the United States, the overall medical expenditure for fatal falls is estimated to be $754 million [1]. This figure underscores the severe impact of falls on healthcare systems worldwide. Reportedly, 15%–50% of patients suffer fall‐related injuries, out of which 1%–10% are severe, including fractures [2, 3, 4, 5]. This scenario is prevalent in other countries, where 25%–55% of nursing home patients experience falls, leading to significant injuries such as fractures, brain haemorrhage or even death, as seen in 1.2%–16.2% of cases in South Korea being fatal [6]. These statistics highlight that falls are not mere accidents but significant global risk factors causing severe secondary accidents.

To minimize falls, several tools have been used to assess their risks upon admission. However, these methods are constrained by their dependence on subjective assessments and patients' recall [7, 8, 9, 10]. Furthermore, these tools are limited in their ability to assess the changes in a patient's physical ability, which is directly related to fall probability. The hospitalized patients may experience significant alterations in their biological conditions and often spend most of their time in beds following admission, leading to decreased physical activity and reduced gait speed, which directly increases the risk of falls [11, 12, 13, 14]. However, a notable limitation of conventional tools is their ineffectiveness in dynamically reflecting changes in patient conditions. Therefore, novel objective tools capable of continuous monitoring and adaptation to the evolving physical abilities and conditions of patients are required [15, 16].

In response to this need, numerous studies have explored the use of the Internet of Things (IoT) and sensor‐based systems for continuous monitoring, demonstrating the potential for advancing fall detection research [17, 18]. Among these technologies, the real‐time location system (RTLS) is particularly noteworthy. RTLS enables continuous monitoring through lightweight sensors embedded in patient identification bands, functioning without the need for charging or additional personal devices, thereby facilitating medical applications such as infection path tracking [19]. The ability of RTLS to continuously collect time‐based location data allows for the objective tracking of changes in patient movements and physical activity. Considering that indicators such as the amount of movement, gait speed and degree of active movement are directly related to falls [13, 14], RTLS provides a valuable means of monitoring these variables.

Additionally, electronic medical records (EMRs) store a wealth of objective information on medical conditions that may reflect a patient's vulnerability. Considering the traceability of RTLS for physical ability and objective information about clinical situations contained in EMR, this study is aimed at overcoming the aforementioned issues by developing a fall‐prediction machine‐learning model. This integrated approach develops a comprehensive and innovative fall‐prediction tool that offers an advanced method for enhancing patient safety and healthcare management.

## 2 Methods

### 2.1 Study Participants and Data Source

We conducted a retrospective study by extracting the EMR and RTLS data from 22 201 patients admitted to Yongin Severance Hospital between 1 March 2020 and 30 June 2022. The patients were provided with RTLS‐equipped wristbands (Figure S1). The RTLS was designed to update its database only when there was a change in the patient's location, capturing the date, time, latitude and longitude. All patients admitted to Yongin Severance Hospital provided broad consent for the collection and use of their location data via RTLS at the time of admission. The study was approved by the Institutional Review Board of Yonsei University Severance Hospital (No. 9‐2021‐0037), with a waiver of additional written consent owing to its retrospective design. All clinical and RTLS data used in this study were fully anonymized, ensuring patient privacy and compliance with data security protocols.

**FIGURE 1:**
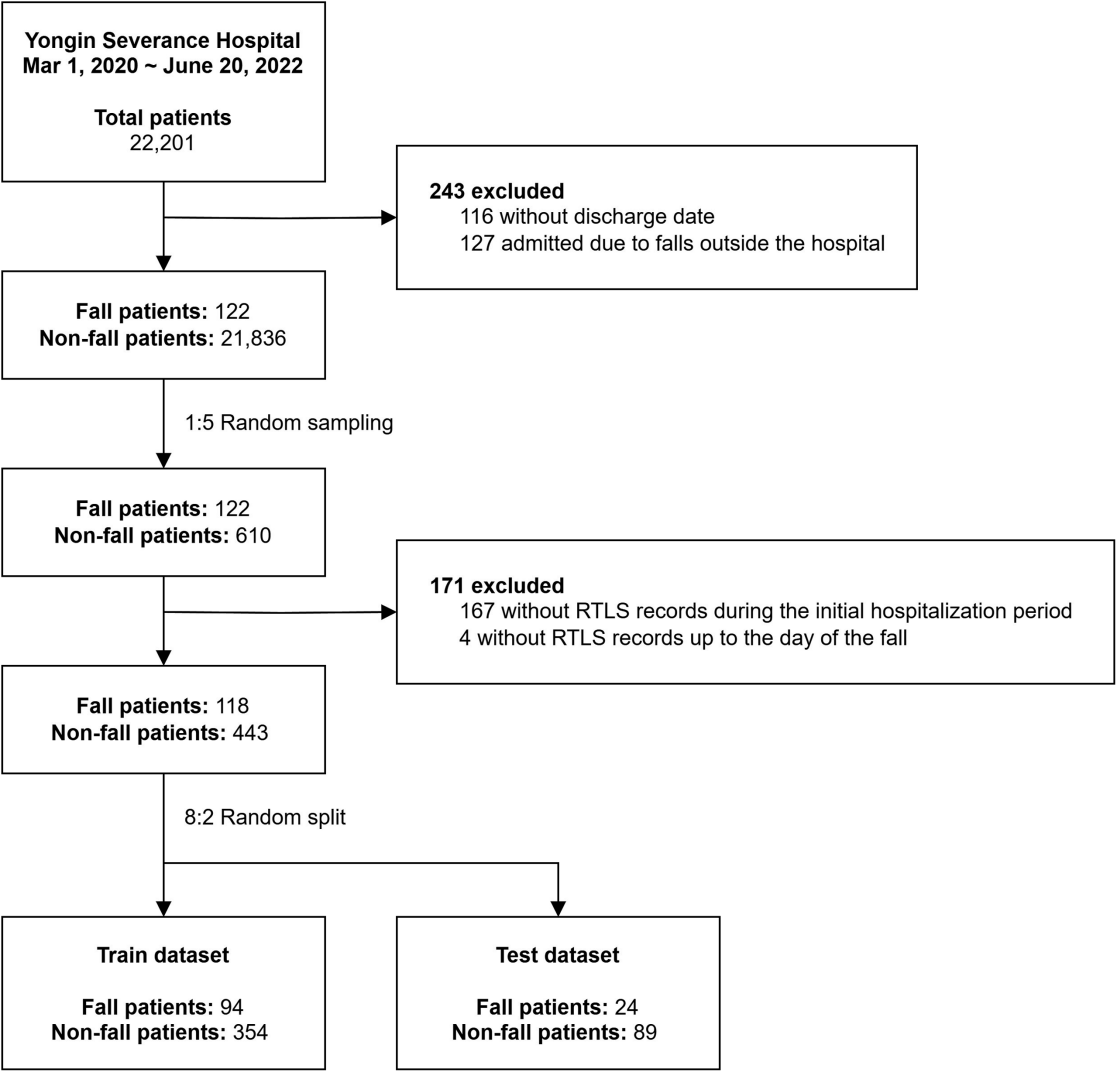
Patient selection flowchart. RTLS: real‐time location system.

The initial cohort of patients was refined by excluding individuals with unavailable discharge records (*n* = 116) and those admitted because of falls from external sources (*n* = 127), thereby focusing our analysis on in‐hospital falls. The patient cohort was divided into the following two groups: those who experienced falls (fall group) and those who did not (nonfall group). To assess the impact of various factors on fall occurrence, we excluded the cases of recurrent falls and focused our analysis solely on the first fall. Subsequently, a random sampling approach was employed to balance the representations of both patient groups. The final stage of cohort refinement involved excluding the patients based on the completeness of the RTLS data. Patients were excluded if their RTLS records were unavailable throughout their hospitalization (*n* = 167) or the RTLS data of fall patients were not recorded prior to the fall event (*n* = 4). These patients were excluded because of incomplete RTLS data, which made it unfeasible to engineer the necessary RTLS features for analysis. After establishing this refined cohort, we applied an 8:2 random split to separate the data into training and testing datasets (Figure 1). An overview of this study is provided in detail in Figure S2.

### 2.2 Features Engineering Using Clinical Information Extracted From EMR

We collected all clinical data routinely assessed in most admitted patients, including anthropometric parameters, vital signs and basic biochemical measurements. From this dataset, we selected variables that were available for at least 70% of patients, resulting in 27 clinical variables included in the model. We focused on the initial EMR data of all admitted patients, including the standard set of measurements performed during admission, considering the anthropometric variables, such as height, weight, systolic blood pressure (SBP), diastolic blood pressure (DBP) and biochemical parameters including glucose, creatinine, alkaline phosphatase (ALP), alanine aminotransferase (ALT), aspartate aminotransferase (AST), bilirubin, blood urea nitrogen (BUN), calcium and lipid profiles and red cell distribution width (RDW). The initial value was selected for model development in the case of repeated measurements. Hospitalization variables, such as the length of stay (LOS) and presence of intensive care unit (ICU) care, were also determined. For nonfall patients, the study period was from admission to discharge, whereas for fall patients, it was from admission to the day of the fall. Additionally, we considered the dosage of two types of drugs—peridol and sedatives—owing to their influence on the movement of patients, resulting in 27 attributes. The department codes were categorized into four types based on the medical department characteristics, as outlined in Table S1. Descriptions and abbreviations of EMR features are provided in Table S2.

### 2.3 RTLS‐Assisted Feature Engineering

Given that gait speed and the degree of active movement are associated with fall risk [13, 14], we explored and calculated a comprehensive range of variables to reflect patients' amount of movement, gait speed and degree of active movement. These variables were derived from daily RTLS data, capturing the first and last days of hospitalization. We also calculated both average and median values to assess movement patterns throughout the stay. Consequently, we derived 28 RTLS variables, all of which were incorporated into the model development.

**FIGURE 2:**
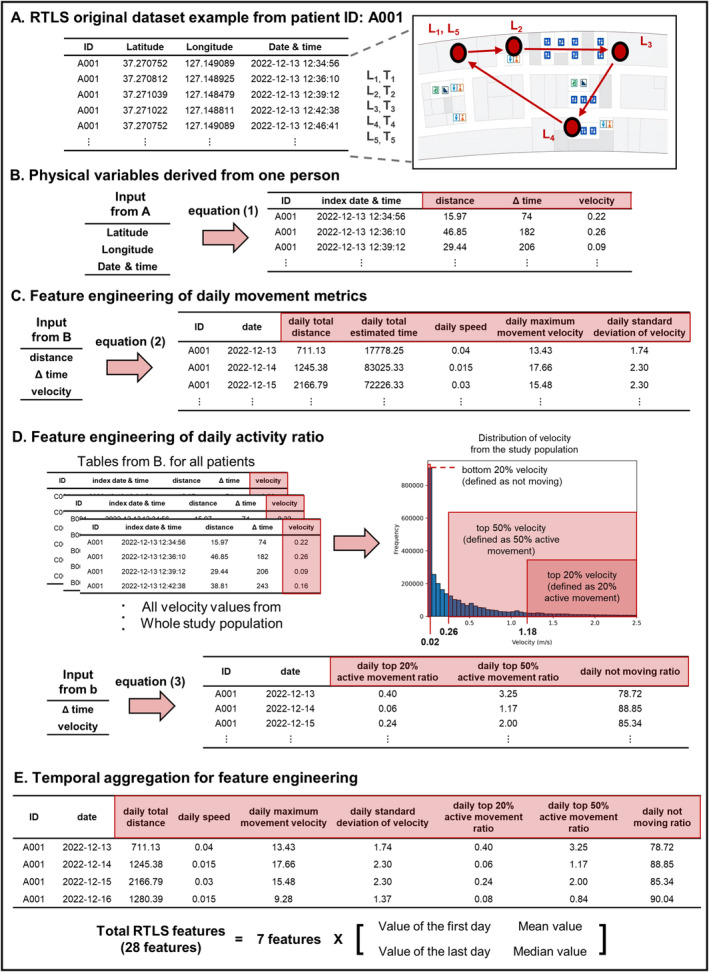
Overall flow of RTLS feature engineering. (A) Original RTLS dataset from patient ID A001. Example of a virtual patient's movements across five locations within the facility (L1 to L5) tracked by the RTLS dataset following the sequence indicated by arrows. (B) Physical variables derived from an individual. Calculation process for distance, Δ time and velocity using the latitude, longitude, date and time via Equation (1) (1.1–1.3). (C, D) Feature engineering. (C) Feature engineering of daily movement metrics using distance, Δ time and velocity to engineer daily total distance, daily total estimated time, daily speed, daily maximum movement velocity and daily standard deviation of velocity using Equation 2 (2.1–2.5), respectively. (D) Feature engineering of the daily activity ratio. Engineering the daily top 20% active movement ratio, daily top 50% active movement ratio and daily not moving ratio using Δ time and velocity via Equation 3 (3.1–3.3). (E) Temporal aggregation for feature engineering. Aggregation of seven core features into 28 RTLS features by calculating the first and last day values, along with the mean and median values for each feature.

Variables from the RTLS records were formulated to measure the dynamics of daily patient movements, as shown in Figure 2. Figure 2A illustrates an example of the RTLS dataset for a hypothetical patient, where *L* and *T* represent the recorded location of the patient and time point, respectively. This dataset serves as the foundation for subsequent feature engineering steps. In all equations, *k* denotes the *k* ^th^ arbitrary row for each point of the same patient and *n* denotes the *n* ^th^ arbitrary day for each patient.

The differences between the consecutive rows in the dataset were initially calculated. The earlier date and time between two consecutive rows referred to as the index date and time, respectively, served as the reference points. Here, Lat_*k*_ and Lng_*k*_ denote the latitude and longitude at location *L*_*k*_, respectively. Consequently, the variation in distance was redefined using the haversine formula, considering the earth's radius (*r*) as 6371 km (1.1). This formula accounts for the curvature of the earth and provides a more accurate measurement of the distance between two points [20]. The change in time was defined as Δ time (1.2). The ratio of these two values is defined as velocity, which represents a vector incorporating the magnitude and direction of movement (1.3). The results presented in Figure 2B were derived using these computations. For calculation, the units of distance and time were meters and seconds, respectively.

(1.1) $$\begin{array}{c} \begin{array}{c} \begin{array}{c} {\text{distance}}_{k}=2r\arcsin\\ \left(\sqrt{\sin^{2}\left(\frac{{\mathrm{Lat}}_{k+1}-{\mathrm{Lat}}_{k}}{2}\right)+\cos\left({\mathrm{Lng}}_{k}\right)\cos\left({\mathrm{Lng}}_{k+1}\right)\sin^{2}\left(\frac{{\mathrm{Lat}}_{k+1}-{\mathrm{Lat}}_{k}}{2}\right)}\;\right) \end{array} \end{array} \end{array}$$

(1.2) $$\Delta \;{\text{time}}_{k}=T_{k+1}-T_{k}$$

(1.3) $${\text{velocity}}_{k}=\frac{{\text{distance}}_{k}}{\Delta \;{\text{time}}_{k}}$$

Subsequently, the basic metrics were aggregated to create features. The sums of daily distance and Δ time were defined as the daily total distance (2.1) and daily total estimated time (2.2), respectively. The ratio of these two sums was defined as the daily speed (2.3). Additionally, the daily maximum velocity and standard deviation were defined as the daily maximum movement velocity (2.4) and daily standard deviation of the velocity (2.5), respectively; the latter was used to examine fluctuations in patient movements [21]. These calculations yielded the results presented in Figure 2C. After excluding the total daily estimated time, the following four daily movement metrics were formulated:

(2.1) $$\text{daily}\;{\text{total distance}}_{n\;\mathrm{th}\;\mathrm{day}}=\sum_{k\in n\;\mathrm{th}\;\mathrm{day}}\left({\text{distance}}_{k}\right)$$

(2.2) $$\text{daily total}\;{\text{estimated time}}_{n\;\mathrm{th}\;\mathrm{day}}=\sum_{k\in n\;\mathrm{th}\;\mathrm{day}}\left(\Delta \;{\text{time}}_{k}\right)$$

(2.3) $${\text{daily speed}}_{n\;\mathrm{th}\;\mathrm{day}}=\frac{\text{daily}\;{\text{total distance}}_{n\;\mathrm{th}\;\mathrm{day}}}{\text{daily total}\;{\text{estimated time}}_{n\;\mathrm{th}\;\mathrm{day}}}$$

(2.4) $$\begin{array}{c} \text{daily maximum}\;{\text{movement velocity}}_{n\;\mathrm{th}\;\mathrm{day}}\\ ={\mathrm{MAX}}_{n\;\mathrm{th}\;\mathrm{day}}\left({\text{velocity}}_{k}\right) \end{array}$$

(2.5) $$\text{daily standard deviation}\;{\text{of velocity}}_{n\;\mathrm{th}\;\mathrm{day}}=\sigma_{n\;\mathrm{th}\;\mathrm{day}}\left({\text{velocity}}_{k}\right)$$

From the velocity data of the entire study population, we analysed the distribution of all velocities and identified the following three significant thresholds: the top 20% of velocities at 1.18 m/s, corresponding to the normal walking speed of adults [22]; median velocity at 0.26 m/s and lower 20% of velocities at 0.02 m/s, representing minimal movement. We calculated the duration for which each patient moved at a speed above these thresholds. Accordingly, the daily top 20% and 50% active movement ratios were defined as the duration for which each patient moved at a speed above 1.18 (3.1) and 0.26 m/s (3.2), respectively. The daily not moving ratio (3.3) was defined as the duration for which each patient moved at a speed below 0.02 m/s, indicating minimal or no movement. The calculation results are shown in Figure 2D. The following three daily activity ratios were engineered based on the velocity thresholds:

(3.1) $$\begin{array}{c} \text{daily}\;\mathrm{top}\;20\%\text{active}\;{\text{movement ratio}}_{n\;\mathrm{th}\;\mathrm{day}}=\\ \frac{\Sigma_{k:{\text{velocity}}_{k}\geq 1.18}\left(\Delta \;{\text{time}}_{k}\right)}{\text{daily total}\;{\text{estimated time}}_{n\;\mathrm{th}\;\mathrm{day}}}\times 100 \end{array}$$

(3.2) $$\begin{array}{c} \text{daily}\;\mathrm{top}\;50\%\text{active}\;{\text{movement ratio}}_{n\;\mathrm{th}\;\mathrm{day}}=\\ \frac{\Sigma_{k:{\text{velocity}}_{k}\geq 0.26}\left(\Delta \;{\text{time}}_{k}\right)}{\text{daily total}\;{\text{estimated time}}_{n\;\mathrm{th}\;\mathrm{day}}}\times 100 \end{array}$$

(3.3) $$\begin{array}{c} \text{daily not}\;{\text{moving ratio}}_{n\;\mathrm{th}\;\mathrm{day}}=\\ \frac{\Sigma_{k:{\text{velocity}}_{k}\leq 0.02}\left(\Delta \;{\text{time}}_{k}\right)}{\text{daily total}\;{\text{estimated time}}_{n\;\mathrm{th}\;\mathrm{day}}}\times 100 \end{array}$$

Finally, these metrics were compiled across multiple days during the stay of each patient (Figure 2E). We included values from the first and last days, as well as the averages and medians of each feature over the duration to demonstrate the dynamics of patient movement. This resulted in a comprehensive set of 28 RTLS‐based features for each patient. Further descriptions of each RTLS feature, including the mobility type (amount of movement, gait speed or degree of active movement) each feature is considered relevant to, are summarized in Table S3.

### 2.4 Data Imputation and Development of the Prediction Model

To address the issue of missing data, the *k*‐nearest neighbour imputation (KNN) algorithm was employed as the initial step to effectively impute missing values. Detailed counts of missing values in the EMR data are presented in Table S2. The modelling was performed using the XGBoost algorithm. To further refine the selected model, we incorporated 10‐fold cross‐validation (CV) and GridSearch for hyperparameter optimization. The Youden index was derived from an optimal model to assess the diagnostic ability. Using 27 clinical and 28 RTLS features, we developed the following three distinct models: the first using only clinical features (clinical model), the second using only RTLS features (RTLS model) and the third combining both the feature sets (clinical + RTLS model).

### 2.5 Performance Assessment

To quantify and compare the performances of the three models on the test set, we employed bootstrapping with 1000 iterations to assess the performance at each iteration using various metrics. The performances were compared using the area under the receiver operating characteristic curve (AUROC), the area under the precision‐recall curve (AUPRC) and the Brier scores. Subsequently, a detailed comparison was performed using the Youden index, which encompasses the accuracy, positive predictive value (PPV), sensitivity, specificity and *F*1 score. In addition, subgroup analysis was conducted to calculate AUROC based on age (≤ 65 and > 65 years), sex (male and female), department code (medicine and surgery, with major and minor surgery) and LOS (≤ 7 and > 7 days).

Additionally, we employed decision curve analysis (DCA) to compare the three models and determine clinical utility [23]. The net benefit refers to the balance between the true‐ and false‐positive predictions for assessing the model's predictive accuracy. Furthermore, SHAP values, including SHAP interaction values, were used to identify both the relative importance and interactions of features influencing the three models.

### 2.6 Statistical Analysis

To discern the statistical variations in patient characteristics, the data were first assessed using the Shapiro–Wilk test for normality. For binary attributes, the chi‐square test was used to determine the statistical significance. For continuous variables, a suitable test was determined based on data distribution. The *t*‐test and Mann–Whitney *U* test were applied when the data adhered to a normal and non‐normal distribution, respectively. Pearson's correlation coefficients were calculated to assess the relationships between continuous variables. In comparing continuous variables to categorical variables, the Kruskal–Wallis test was applied to evaluate their associations. DeLong's test was used to compare AUROC across subgroups. The Kruskal–Wallis test also performed a nonparametric comparison of the distributions of outcome values across the three models. Subsequently, a post hoc analysis was performed using the Bonferroni adjustment in conjunction with Dunn's test to compare the significance across specific subgroups. Different levels of statistical significance are denoted in the plots as follows: an asterisk (*) for *p*‐values less than 0.05, double asterisks (**) for *p*‐values < 0.01 and triple asterisks (***) for *p*‐values < 0.001.

## 3 Results

### 3.1 Baseline Clinical Characteristics

Before analysis, 171 patients were excluded because of incomplete RTLS data, which would have affected the integrity of the RTLS‐based feature extraction. The study cohort finally comprised 118 patients with falls and 443 patients without falls (Figure 1). Statistical comparison of baseline characteristics between fall and nonfall groups revealed significant differences in several clinical and RTLS features (Table 1 and Table S4). To further illustrate these differences, the distribution of clinical variables is presented in Figure S3 and RTLS features in Figure S4.

**TABLE 1:** Baseline characteristics of patients.

	Overall (*n* = 561)	Fall (*n* = 118)	Nonfall (*n* = 443)	*p*
Sex				0.003
Female	266 (47.4%)	41 (34.7%)	225 (50.8%)	
Male	295 (52.6%)	77 (65.3%)	218 (49.2%)	
Age (years)	71.0 [60.0–80.0]	73.0 [65.0–81.0]	70.0 [59.0–79.0]	0.014
LOS (days)	5.0 [3.0–11.0]	7.5 [3.0–16.0]	5.0 [2.0–9.0]	0.003
Department code				0.019
Medicine	335 (59.7%)	81 (68.6%)	254 (57.3%)	
Major surgery	167 (29.8%)	29 (24.6%)	138 (31.2%)	
Minor surgery	58 (10.3%)	7 (5.9%)	51 (11.5%)	
Others	1 (0.2%)	1 (0.8%)	0 (0.0%)	
ICU admission	35 (6.2%)	13 (11.0%)	22 (5.0%)	0.028
SBP (mmHg)	128.6 (21.5)	128.7 (23.6)	128.5 (21.0)	0.946
DBP (mmHg)	77.2 (13.8)	77.2 (13.6)	77.2 (13.9)	0.999
Pulse rate (/min)	75.7 (15.5)	79.0 (15.1)	74.8 (15.6)	0.009
BMI (kg/m^2^)	23.7 (3.9)	23.0 (3.9)	23.8 (3.8)	0.04
Sedative	126 (22.5%)	51 (43.2%)	75 (16.9%)	< 0.001
Peridol	42 (7.5%)	15 (12.7%)	27 (6.1%)	0.026
Albumin (g/dL)	3.9 (0.6)	3.8 (0.6)	3.9 (0.7)	0.137
ALP (IU/L)	97.5 (86.9)	98.7 (76.4)	97.1 (90.2)	0.853
ALT (IU/L)	31.8 (75.6)	24.6 (24.0)	34.1 (85.7)	0.058
AST (IU/L)	40.6 (60.7)	37.1 (36.3)	41.8 (66.7)	0.336
Total bilirubin (mg/dL)	0.9 (1.6)	0.9 (1.6)	0.9 (1.6)	0.802
BUN (mg/dL)	22.1 (17.4)	23.4 (15.6)	21.7 (17.9)	0.324
Calcium (mg/dL)	8.7 (0.7)	8.7 (0.7)	8.7 (0.6)	0.875
Total cholesterol (mg/dL)	150.6 (45.9)	145.8 (45.3)	152.2 (46.1)	0.187
Creatinine (mg/dL)	1.3 (1.8)	1.5 (2.1)	1.2 (1.7)	0.181
Glucose (mg/dL)	141.4 (74.1)	149.2 (77.1)	138.9 (73.1)	0.205
HCT (%)	36.5 (6.8)	35.5 (6.7)	36.9 (6.8)	0.059
Haemoglobin (g/dL)	12.2 (2.4)	11.9 (2.4)	12.3 (2.4)	0.09
Phosphate (mg/dL)	3.4 (1.0)	3.3 (0.8)	3.5 (1.1)	0.053
Total protein (g/dL)	6.4 (0.8)	6.4 (0.8)	6.4 (0.8)	0.474
RDW (%)	13.7 (2.4)	14.4 (2.8)	13.5 (2.2)	0.003
Uric acid (mg/dL)	5.1 (2.2)	5.2 (2.2)	5.0 (2.2)	0.462
Average of daily total distance (m)	4610.1 [2821.7–6530.0]	4410.2 [2586.5–5979.2]	4683.6 [2832.7–6640.5]	0.225
Average of daily speed (m/s)	0.07 [0.05–0.11]	0.06 [0.04–0.09]	0.08 [0.05–0.11]	0.023
Average of daily max velocity (m/s)	20.3 [14.2–31.2]	19.9 [14.5–29.3]	20.4 [14.2–32.3]	0.441
Average of daily standard deviation of velocity (m/s)	2.2 [1.7–3.2]	2.3 [1.6–3.2]	2.2 [1.7–3.2]	0.854

*Note:* Data are presented as median (IQR) for non‐normally distributed variables, mean (SD) for normally distributed variables and *n* (%) for categorical variables. Units are specified next to each variable. The Shapiro–Wilk test was used to evaluate the normality of continuous variables. For binary attributes, the statistical significance was assessed using the chi‐square test. For nonbinary continuous variables, the *t*‐test was employed to meet the normality criteria, whereas the Mann–Whitney *U* test was applied to data not adhering to a normal distribution.

Abbreviations: ALP: alkaline phosphatase; ALT: alanine aminotransferase; AST: aspartate aminotransferase; BMI: body mass index; BUN: blood urea nitrogen; DBP: diastolic blood pressure; HCT: haematocrit; ICU: intensive care unit; LOS: length of stay; RDW: red blood cell distribution width; SBP: systolic blood pressure.

In Table 1, patients experiencing falls were predominantly older males with longer LOS and a higher proportion of patients using sedatives or peridol (*p* < 0.05 for all). They were primarily admitted for medical treatment and exhibited higher rates of ICU admission (*p* < 0.05 for all). Regarding biochemical parameters, there were no significant differences between the groups for most variables; however, the RDW values were notably higher in the fall group than in the nonfall group (*p* = 0.003). The RTLS data revealed significant disparities in the daily speed on the last day, as well as in the mean and median daily speed, indicating a slower daily speed in the fall group than in the nonfall group during LOS (*p* < 0.05 for all) (Table S4). In addition, it was found that for the daily top 20% movement ratio, there were notable differences not only in the average and median values throughout their LOS but also in the values recorded on the last day between the groups, indicating a lower proportion of daily top 20% movement ratio in fall groups. Similarly, significant differences were also observed for the daily top 50% movement on the last day and in its average value between the groups (Table S4). These underscore a marked reduction in overall physical activity levels in the fall group compared to the nonfall group throughout their LOS.

### 3.2 Performance Comparison of the Three Models for Fall Prediction

**FIGURE 3:**
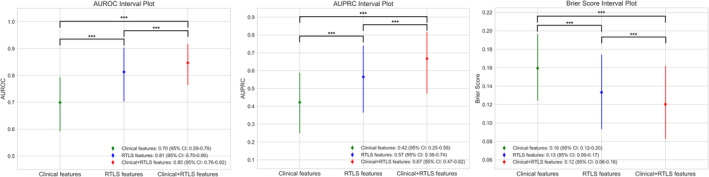
Graphical performance evaluation of the three models. Performance comparison of the three models using AUROC, AUPRC and Brier score metrics based on 1000 bootstrap resampling. Results are presented with 95% confidence intervals. Statistical significance was assessed using the Kruskal–Wallis test, followed by Dunn's post hoc test, with significance levels indicated on the graph (*p < 0.05, **p < 0.01 and ***p < 0.001). The clinical, RTLS and clinical + RTLS models are denoted in green, blue and red, respectively. AUROC: area under the receiver operating characteristics curve; AUPRC: area under the precision recall curve; CI: confidence interval.

The performances of the three models derived from the patient cohorts were compared using the test set. The RTLS model demonstrated robust predictive capability for in‐hospital falls, achieving an AUROC of 0.813 (95% CI: 0.703–0.903). This indicates a superior performance compared to the model using only clinical variables, which had an AUROC of 0.699 (95% CI: 0.592–0.794). Furthermore, when the RTLS model was combined with clinical variables, it exhibited an enhanced AUROC of 0.847 (95% CI: 0.764–0.917). This was significantly higher than the models using either clinical variables alone or the RTLS model alone, thereby revealing statistically significant improvements in performance by integrating comprehensive variables. Similarly, the clinical, RTLS and clinical + RTLS models achieved AUPRC values of 0.423 (95% CI: 0.250–0.590), 0.565 (95% CI: 0.365–0.741) and 0.667 (95% CI: 0.472–0.816), respectively, demonstrating parallel significant differences. The corresponding Brier scores for the models were 0.159 (95% CI: 0.124–0.196), 0.133 (95% CI: 0.093–0.174) and 0.120 (95% CI: 0.083–0.162), with the clinical + RTLS model exhibiting the best performance (Figure 3 and Table 2). Based on other metrics for comparing model performances, the clinical + RTLS model outperformed the other models, exhibiting statistically significant variations and achieving an accuracy of 0.848 (95% CI: 0.770–0.912), PPV of 0.681 (95% CI: 0.462–0.889), sensitivity of 0.540 (95% CI: 0.320–0.750), specificity of 0.931 (95% CI: 0.872–0.978) and *F*1 score of 0.600 (95% CI: 0.390–0.759) (Figure S5 and Table S5).

**TABLE 2:** Comparative performances of the three models.

Model	Youden index	AUROC	AUPRC	Brier score
Clinical model	0.298	0.699 (0.592–0.794)	0.423 (0.250–0.590)	0.159 (0.124–0.196)
RTLS model	0.428	0.813 (0.703–0.903)	0.565 (0.365–0.741)	0.133 (0.093–0.174)
Clinical + RTLS model	0.376	0.847 (0.764–0.917)	0.667 (0.472–0.816)	0.120 (0.083–0.162)

*Note:* Displayed values are the performance metrics for the clinical, RTLS and clinical + RTLS models, with 95% confidence intervals (CIs) in parentheses.

Abbreviations: AUPR: area under the precision‐recall curve; AUROC: area under the receiver operating characteristic curve; RTLS: real‐time location system.

### 3.3 Comparison of the Model Feature Importance Between the Three Models

**FIGURE 4:**
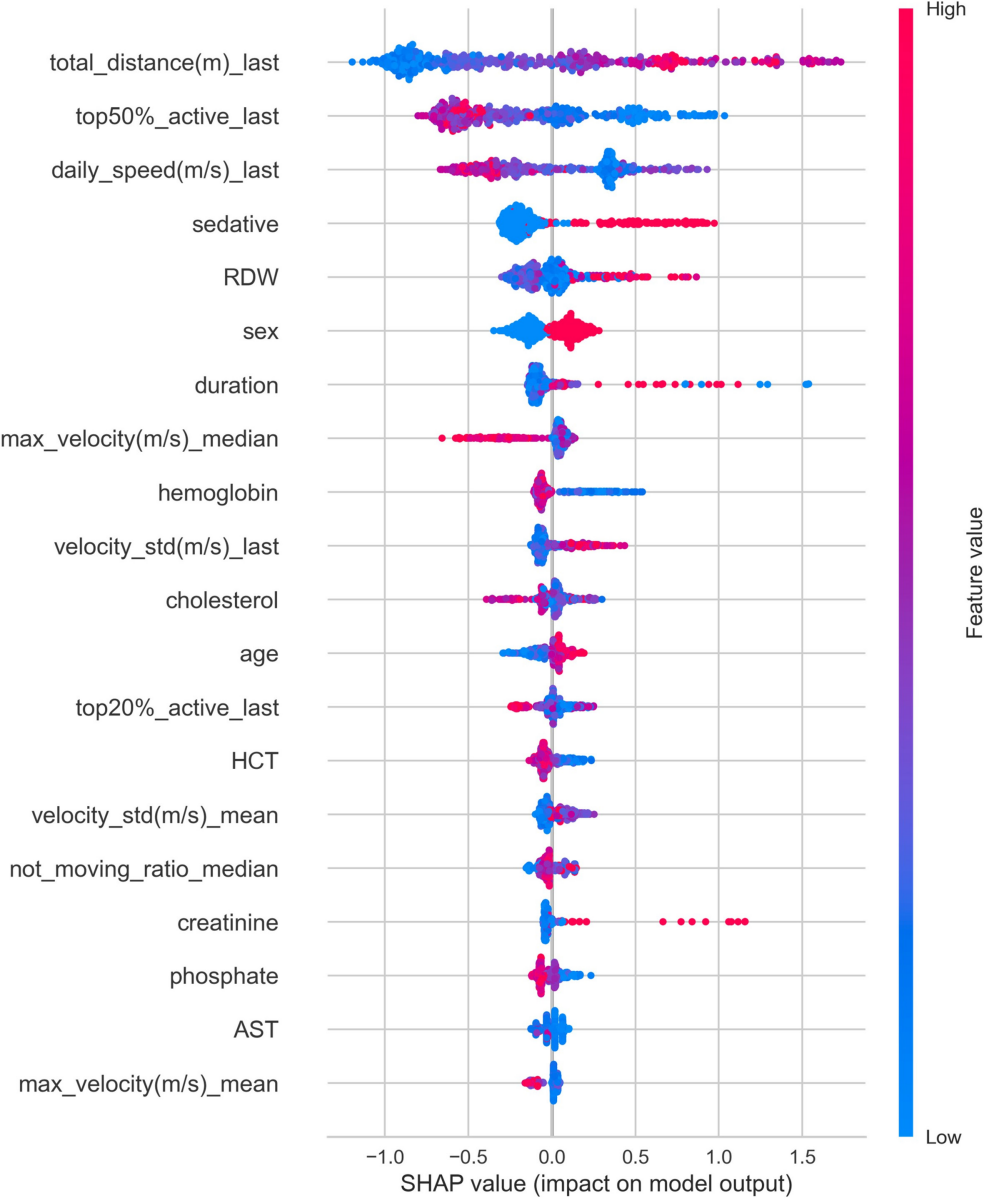
Visualizing feature importance for the combined clinical + RTLS model using SHAP values. Impact of the Top 20 features on model prediction, ranked based on SHAP values. The *y*‐axis displays features in the order of decreasing impact, whereas the *x*‐axis quantifies their SHAP values, with the colour intensity ranging from blue for lower values to red for higher values. total_distance(m)_last: total distance moved (in meter) on the last day of RTLS recording; top50%_active_last: ratio of time spent in the top 50% of active movements on the last day of RTLS recording; max_velocity(m/s)_median: median of daily maximum velocities recorded throughout hospitalization; velocity_std(m/s)_last: standard deviation of velocities on the last day of RTLS recording; top20%_active_last: ratio of time spent in the top 20% of active movements on the last day of RTLS recording; velocity_std(m/s)_mean: mean of daily velocity standard deviations throughout hospitalization; not_moving_ratio_median: median of the daily ratios of time spent without movement throughout hospitalization; max_velocity(m/s)_mean: mean of daily maximum velocities recorded throughout hospitalization; LOS: length of stay; AST: aspartate aminotransferase; HCT: haematocrit, RDW: red cell distribution width; SHAP value: Shapley additive explanations value.

Figure 4 shows the feature importance of the clinical + RTLS model, which exhibited the best performance. The significance of each feature was assessed and compared across the remaining models (Figure S6). The predominant features influencing the clinical model (Figure S6A) and the RTLS feature model (Figure S6B) were largely retained in the clinical + RTLS model.

In the clinical + RTLS model, SHAP analysis identified the daily total distance moved (total_distance), the daily top 50% active movement ratio (top50%_active) and the daily movement speed (daily_speed) as the top three ranked features, with their values on the last day of hospitalization being particularly impactful (Figure 4). It was found that a greater total distance moved on the last day significantly increased the likelihood of a fall occurring. Conversely, a lower daily top 50% active movement ratio and a slower daily movement speed on the last day were found to be strongly predictive of falls. A greater total distance moved on the last day significantly increased the likelihood of a fall occurring. Conversely, a lower daily top 50% active movement ratio and a slower daily movement speed on the last day were found to be strongly predictive of falls. Furthermore, the fourth‐ and fifth‐ranked features revealed that the elevated RDW levels of patients who were administered sedative drugs greatly affected the fall incidents.

### 3.4 Comparative Evaluation of the Model Clinical Efficacy Using Net Benefit Analysis

**FIGURE 5:**
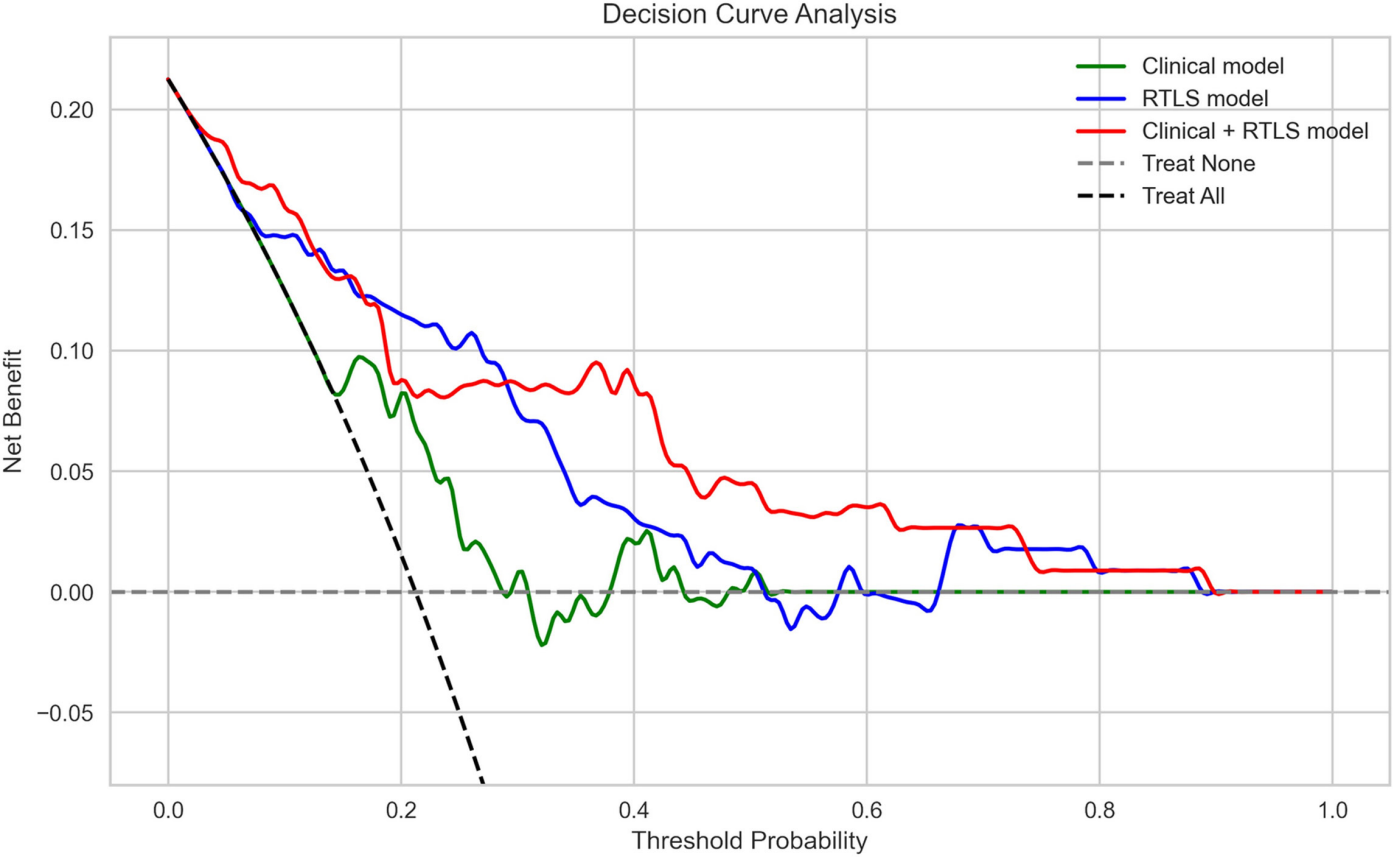
DCA of each model performance. Decision curve analysis comparing the models based on net benefit across decision thresholds. Net benefit quantifies the trade‐off between the benefit of true positives and harm of false positives. The clinical, RTLS and clinical + RTLS models are denoted in green, blue and red, respectively. RTLS: real‐time location system.

A result of DCA comparing the three models revealed that the clinical model, which utilized only EMR variables, exhibited the lowest net benefit and offered minimal clinical advantages (Figure 5). Contrastingly, the clinical + RTLS model, consistent with its performance in other metrics, exhibited a superior net benefit across various thresholds compared with the other two models.

### 3.5 Feature Interaction and Subgroup Analysis

The analysis of interactions between clinical and RTLS variables showed that among the numerous combinations of 27 clinical and 28 RTLS variables, age and department code emerged as top‐ranking variables when selecting the Top 6 based on Pearson's correlation and Kruskal–Wallis tests. Correlations with age included daily total distance (average over LOS) at −0.292, daily top 20% active movement ratio (median over LOS) at −0.330 and daily speed (median over LOS) at −0.310, all with *p*‐values below 0.001 (Figure S7). The Kruskal–Wallis test indicated significant differences between department code and RTLS features, with the largest differences observed in the daily top 20% active movement ratio (average over LOS) (*H* = 17.075, *p* < 0.001), median over LOS (*H* = 14.666, *p* < 0.002) and daily top 20% active movement ratio on the first day (*H* = 14.040, *p* < 0.003). Based on these Top 6 combinations, SHAP interaction values were used to further evaluate the interaction between clinical and RTLS features in predicting fall risk (Figure S8). The results indicated an insignificant interaction between the two variable types, as shown in the interaction plots, suggesting that clinical and RTLS features contributed independently to the model's predictions.

In the subgroup analysis, performance across the three models was compared based on age, sex, department code and LOS (Table S6), and no statistically significant differences in AUROC were observed among the subgroups, including in the clinical + RTLS model. This indicates consistent predictive capability across all subgroups without significant variations.

## 4 Discussion

The results of this study indicate a significant advancement in the field of fall prediction during hospitalization by utilizing a novel approach that integrates the RTLS variables capturing patient movement with the clinical variables obtained from EMR. The RTLS model alone showed a strong ability to predict in‐hospital falls, achieving an AUROC of 0.813 (95% CI: 0.703–0.903), an AUPRC of 0.565 (95% CI: 0.365–0.741) and a Brier score of 0.133 (95% CI: 0.093–0.174). When combined with clinical data, the clinical + RTLS model achieved an AUROC of 0.847 (95% CI: 0.764–0.917), an AUPRC of 0.667 (95% CI: 0.472–0.816) and a Brier score of 0.120 (95% CI: 0.083–0.162), significantly outperforming the models using either dataset alone. The clinical benefits of the proposed model are further confirmed by DCA. These findings may significantly enhance fall prevention by facilitating a more convenient and precise prediction of such incidents.

By utilizing real‐time data from RTLS, we engineered variables capturing variations in patient movement and incorporated initial clinical scores and medication histories from EMRs to enhance fall prediction performance. Instead of relying on a single model, this study simultaneously presents three independently developed models based on clinical features, RTLS data and a combination of these two. Each dataset facilitates commendable predictive performance individually; however, the integrated model demonstrates statistically significant superiority, indicating that the initial clinical measurements and RTLS‐derived features contribute to prediction accuracy. The model's consistent performance across various subgroups did not show any statistically significant variation, supporting its stable predictive capability under different conditions.

The proposed approach remarkably overcomes the limitations of conventional methods that lack the ability to continuously and objectively measure patient movement [7, 8, 9, 24, 25]. Furthermore, several studies have shown that machine learning models utilizing variables such as historical fall‐related injuries, gait assessments and various EMR data achieve an AUROC of approximately 0.70–0.88 [26, 27, 28]. Compared to these, our model's performance demonstrates competitive results with an AUROC of 0.847. Another sensor‐based system, such as smart insoles, showed an AUROC of around 0.80, which is comparable to our findings [29]. Smartphones, which require constant GPS activation, pose challenges in hospital management as patients may turn off GPS, and additional operations on personal devices might be undesirable for patients. Devices like wristwatches or smart insoles necessitate regular charging and can be cumbersome for patients, as well as being cost‐prohibitive. In contrast, RTLS employs lightweight sensors embedded in patient identification bands, eliminating the need for charging and additional wear. RTLS has significant potential in patient safety management, with established applications in infection control studies, such as monitoring COVID‐19 spread within hospitals [19]. Additionally, continuous tracking of patient movement and location can help mitigate risks associated with in‐hospital conditions such as delirium, which may arise from prolonged inactivity or isolation. This automated patient monitoring significantly reduces the workload traditionally required for close and frequent observation of high‐risk patients, thereby easing the demands on healthcare staff. Although some workload remains for system management, such as maintaining sensors and data management to ensure quality and completeness, the shift from manual to automated monitoring could reduce staff burden. The extent of this benefit and the balance between workload reduction and system and data management challenges warrant further discussion and investigation to better understand the practical value of RTLS. Despite these challenges, RTLS remains a valuable tool for enhancing real‐time patient monitoring and improving operational efficiency. Using this RTLS‐based system, we calculated patient movements and developed a model with satisfactory performance using simple initial clinical metrics and medication histories, without relying on extensive medical records. This indicates that RTLS provides a practical and efficient solution for continuous patient monitoring compared to the aforementioned methods.

SHAP analysis provides insights into the most influential features of the high‐performing clinical + RTLS model. Among the Top 5 features, three were derived from RTLS (daily total distance, daily speed and daily top 50% active movement ratio), and the remaining two were derived from the EMR data, indicating a balanced contribution from the two data sources. The RTLS variables, particularly the daily total distance, represent the total amount of patient movement in a day. Contrastingly, the daily speed represents an average daily measure of the gait speed, a well‐known factor associated with falls that are emphasized in conventional methods [13, 30, 31, 32]. The daily top 50% active movement ratio, which indicates the proportion of time spent moving at a faster pace, serves as an indirect measure of physical activity, another key factor in determining fall risk [11, 33]. These RTLS variables are analysed across various time points, including initial hospitalization and the day of the fall, revealing that the values on the last day of stay are particularly important. Therefore, the patient movement patterns closer to the time of fall are more critical than those at the beginning of hospitalization. This aligns with the existing research advocating the continuous monitoring of patients to prevent falls [15, 16]. Hence, a decrease in physical activity and gait speed on days closer to the fall, particularly in patients with more movement, is correlated with an increased risk of falling.

In addition to RTLS variables, high RDW and sedative use are associated with an increased risk of falls, corroborating the findings from existing research. Sedatives, which are central nervous system depressants typically prescribed for anxiety and sleep disorders, are associated with falls owing to their effects such as slowing reaction times and causing dizziness [34, 35]. The relationship between sedative use and fall risk highlights the need for careful management of these medications in hospitalized patients, particularly in those at a higher risk of falls. An increase in this metric may indicate the biological aging process impairing neuromuscular function, which can increase the fall risk as it potentially affects balance, coordination and overall physical strength [33]. The correlation between higher RDW and fall risk indicates that this haematological parameter could serve as a useful biomarker for fall risk assessment. RDW has been known to be closely related to aging phenotypes in diverse clinical situations [36, 37, 38]. Moreover, it is known that males are more likely to experience falls [39]. Our study also found that male sex is significantly associated with an increased risk of falls. Although age was not among the top five features in the SHAP analysis, it was clearly associated with an increased risk of falls as patients aged. Integrating these clinical variables, including sedative use, elevated RDW levels, male sex and older age, with the proposed model, combined with RTLS‐derived variables, provides a more comprehensive understanding of the multifactorial nature of fall risk in hospital settings. Identifying patients who exhibit these patterns allows healthcare providers to take proactive measures in fall prevention, thereby enhancing patient safety and care quality.

In the clinical + RTLS model, the interaction between clinical and RTLS variables was explored using SHAP interaction values for six representative combinations of statistically significant variables. The analysis showed negligible interaction between the two types of variables, suggesting that clinical features and RTLS data contributed independently to the prediction of fall risk. Based on these six combinations, both clinical and RTLS variables offer meaningful contributions without significant interdependence. Although this observation is limited to these specific combinations, it underscores the effectiveness of the combined use of these variables in yielding strong predictive performance. Thus, incorporating both data types is essential for a more comprehensive assessment of fall risk.

The implications of this study can be understood in three ways. First, the model generalizability is emphasized because the study was based on a randomly sampled population of all hospitalized patients in a tertiary care hospital, rather than being limited to specific wards or disease conditions. Second, by capturing the real‐time dynamics of patient movements using RTLS, the proposed model overcomes the limitations of conventional fall prediction methods, offering a more accurate and timely assessment of fall risk. Third, the alignment of the key variables of the model, identified via SHAP analysis, with the reported fall risk factors underscores the strong explainability of the model. This congruence validates the fundamental requirement for clinically verified knowledge, thus enhancing the credibility and utility of the model in clinical settings.

The primary limitation of this study is the requirement for multicohort validation. The proposed approach, involving 1000 iterations with duplication in the test set, serves as a provisional measure to statistically validate the model performance; however, future research should perform validation across diverse cohorts. Another limitation is data imbalance owing to the varying patient event distributions. Despite mitigating this issue via random sampling, disparities in patient data persisted, which may have affected the accuracy and precision of the findings. Additionally, the exclusion of patients with incomplete RTLS data introduced the potential for selection bias. In this study, 171 patients were excluded because of incomplete RTLS data, which would have affected the integrity of the RTLS‐based feature extraction. Among these, 104 were discharged on the same day (LOS = 0), usually following outpatient surgery, providing an insufficient period for monitoring. The remaining 67 patients were younger and had shorter LOS, lower rate of sedative use and lower fall incidence, reflecting a lower risk profile (data not shown). Applying RTLS, it is more feasible to focus on the patient with longer LOS with a higher risk of falls. Therefore, the exclusion of milder cases may not affect the value of RTLS. Nonetheless, the exclusion of these cases may influence the results. Further research should improve system reliability and develop strategies for handling incomplete RTLS data to mitigate this potential bias. The reliance on RTLS and EMR data, while informative, could omit potential environmental or situational factors influencing the fall risk. Further research is necessary to capture these broader aspects and enhance the understanding of fall risk in hospital settings.

Although the combination of RTLS and clinical parameters effectively predicts in‐hospital falls, its implementation may be limited to hospitals without access to such systems. Our findings suggest that patients who take sedatives, exhibit elevated RDW levels, reduce physical activity, have slower gait speed or compensate by moving longer distances during hospitalization are at a higher risk of falls. For these patients, implementing more frequent monitoring and closer observation could enable timely interventions and help reduce fall risk. Building on the RTLS‐based feature engineering methods developed in this study, future research could explore how these approaches can be adapted to clinical environments without RTLS. In such cases, technologies such as standard actigraphy could serve as an alternative for monitoring patient movement and identifying fall risks. Additionally, in nonhospital settings such as homes, wearable devices or smartphones could provide real‐time tracking and alerts, empowering patients to monitor their own fall risk. Further advancements are required to tailor these solutions to various technologies and environments, ensuring that fall prevention strategies can be effectively implemented across diverse clinical and home settings.

To the best of our knowledge, this study is the first to apply RTLS to fall prediction with a machine learning approach. Advancements in IT technologies have enabled the tracking of patient locations within hospitals using RTLS. This study seeks to overcome the limitations of previous research on falls by leveraging technological progress. By utilizing real‐time data on patient location changes, our findings suggest that integrating RTLS with EMR can significantly improve fall prediction accuracy. This IT‐enhanced approach may facilitate the early detection of fall risks during hospitalization, thereby preventing fall incidents, augmenting patient safety and alleviating the workload of healthcare professionals by implementing automated solutions.

## Ethics Statement

This study was approved by the Institutional Review Board of Yonsei University Severance Hospital (No. 9‐2021‐0037). The authors certify that they comply with the ethical guidelines for authorship and publishing in the *Journal of Cachexia, Sarcopenia and Muscle* [40].

## Conflicts of Interest

The authors declare no conflicts of interest.

## Supporting information


**Figure S1.** RTLS sensors of the Yongin Severance Hospital.
**Figure S2.** Overall research flow.
**Figure S3.** Clinical variable distributions by fall status.
**Figure S4.** RTLS variable distributions by fall status.
**Figure S5.** Additional performance evaluation using the interval plots of the three models.
**Figure S6.** Feature importance of clinical and RTLS models using SHAP values.
**Figure S7.** Pearson's correlation matrix of continuous variables.
**Figure S8.** SHAP interaction values between clinical and RTLS features in the clinical + RTLS model.
**Table S1.** Department code classification.
**Table S2.** Descriptions and counts of the missing clinical feature values.
**Table S3.** Descriptions of RTLS features.
**Table S4.** Baseline characteristics (RTLS features) of patients.
**Table S5.** Additional comparative performance metrics of the three models.
**Table S6.** Subgroup analysis of model performance based on specific clinical conditions.
