# Supplementary material for: Predicting In‐Hospital Fall Risk Using Machine Learning With Real‐Time Location System and Electronic Medical Records
Source: J Cachexia Sarcopenia Muscle. 2025 Feb 24;16(1):e13713. doi: 10.1002/jcsm.13713 (PMC11850291; doi:10.1002/jcsm.13713)
Supplement: Supplementary file 1 — Figure S1. RTLS sensors of the Yongin Severance Hospital. Figure S2. Overall research flow. Figure S3. Clinical variable distributions by fall status. Figure S4. RTLS variable distributions by fall status. Figure S5. Additional performance evaluation using the interval plots of the three models. Figure S6. Feature importance of clinical and RTLS models using SHAP values. Figure S7. Pearson's correlation matrix of continuous variables. Figure S8. SHAP interaction values between clinical and RTLS features in the clinical + RTLS model. Table S1. Department code classification. Table S2. Descriptions and counts of the missing clinical feature values. Table S3. Descriptions of RTLS features. Table S4. Baseline characteristics (RTLS features) of patients. Table S5. Additional comparative performance metrics of the three models. Table S6. Subgroup analysis of model performance based on specific clinical conditions. [file JCSM-16-e13713-s001.docx]

**Predicting In-Hospital Fall Risk Using Machine Learning with Real-Time Location System and Electronic Medical Records**

**Supplementary material**

**Table of contents**

***Supplementary Figures***

*Figure S1. RTLS sensors of the Yongin Severance hospital.*

*Figure S2. Overall research flow.*

*Figure S3. Clinical variable distributions by fall status.*

*Figure S4. RTLS variable distributions by fall status.*

*Figure S5. Additional performance evaluation using the interval plots of the three models.*

*Figure S6. Feature importance of clinical and RTLS models using SHAP values.*

*Figure S7. Pearson correlation matrix of continuous variables.*

*Figure S8. SHAP Interaction Values Between Clinical and RTLS Features in the Clinical + RTLS Model.*

***Supplementary Tables***

*Table S1. Department code classification.*

*Table S2. Descriptions and counts of the missing clinical feature values.*

*Table S3. Descriptions of RTLS features.*

*Table S4. Baseline characteristics (RTLS features) of patients.*

*Table S5. Additional comparative performance metrics of the three models.*

*Table S6. Subgroup analysis of model performance based on specific clinical conditions.*


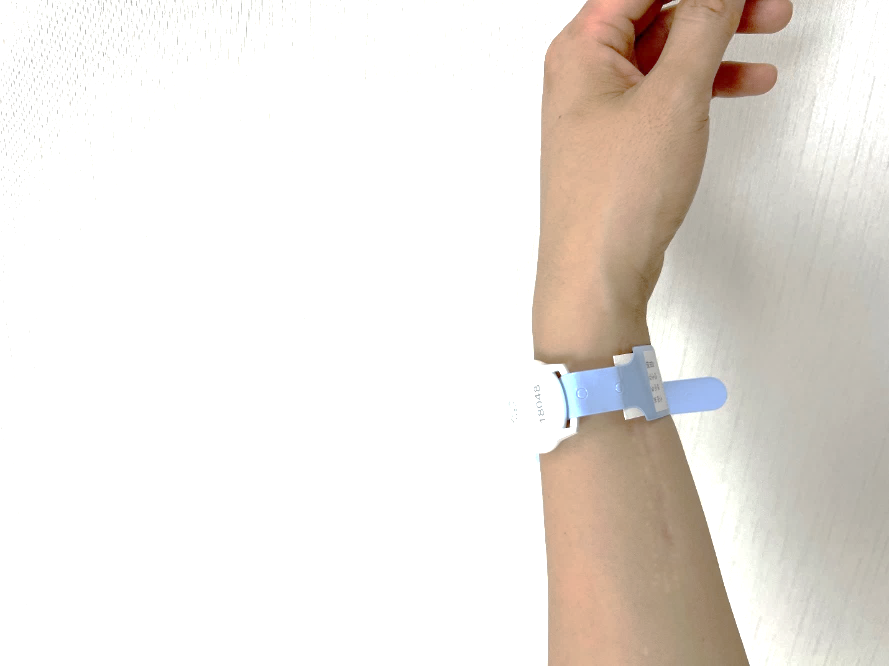
**Figure S1** **RTLS sensors of the Yongin Severance hospital**

**A. RTLS-equipped wristbands of patients**


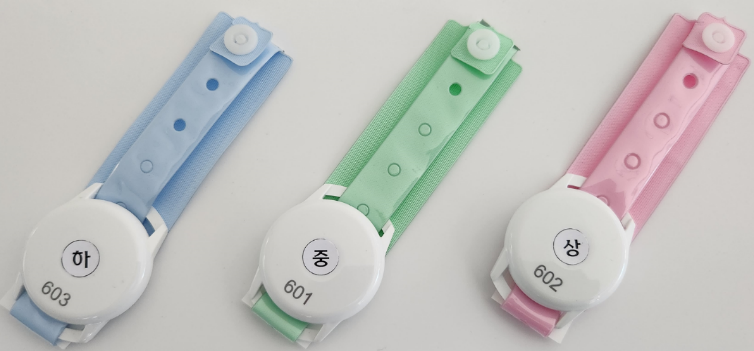


**B. On-model display of RTLS-equipped wristband**


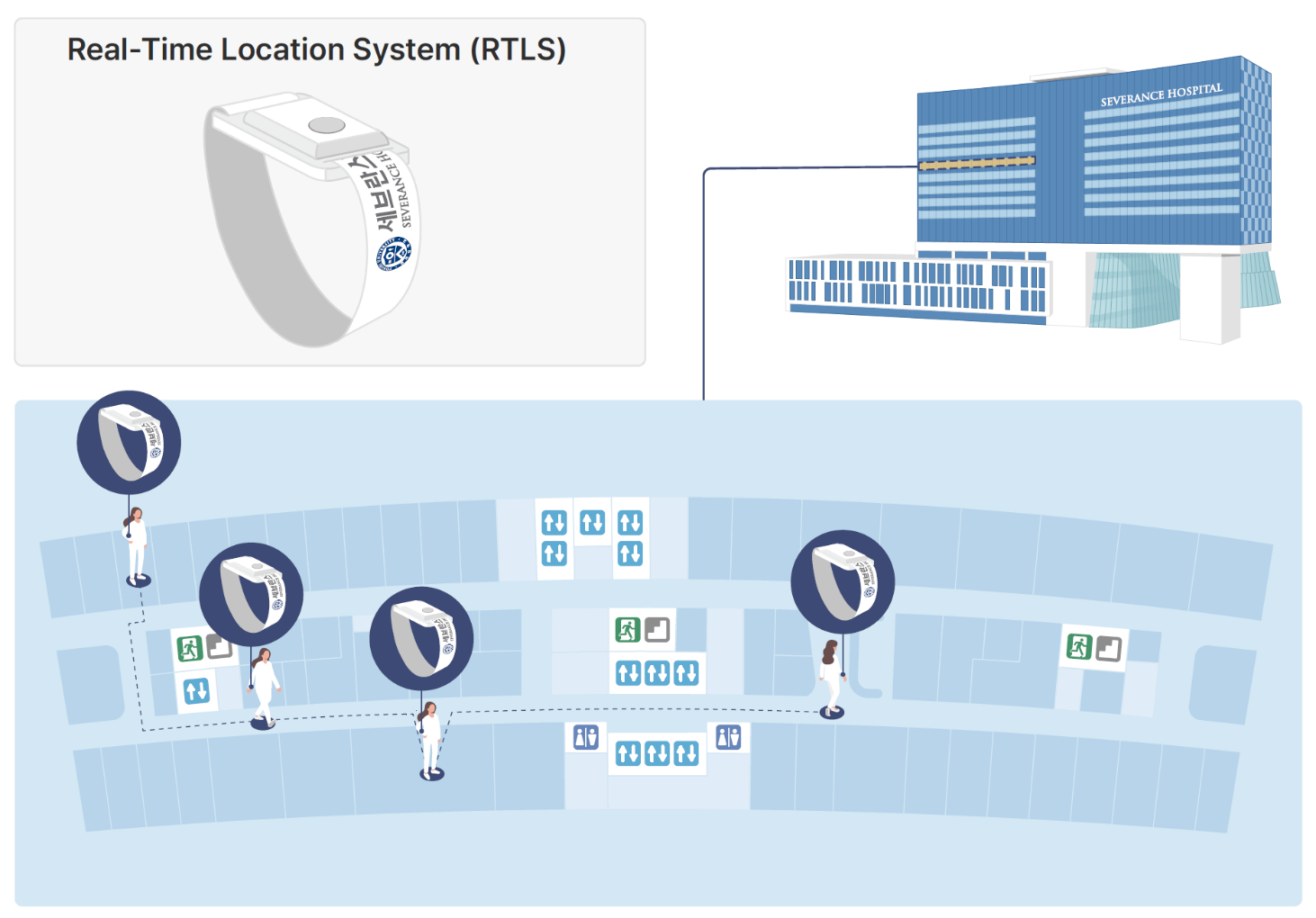


**C. Diagrammatic illustration of RTLS sensor measurement in Yongin Severance hospital**

A, RTLS-equipped wristbands of patients serving as identification bracelets with embedded RTLS sensors. The wristbands are color-coded in blue, green, and red to represent patient risk levels, with each color indicating a different level of severity. B, Photograph of a patient wearing an RTLS-equipped wristband. C, Operational schematic of RTLS sensor tracking at Yongin Severance Hospital. The illustration above depicts a simplified blueprint of Yongin Severance Hospital. Below, the image illustrates the tracking of patient movement within a single floor, as recognized by RTLS wristband sensors. The RTLS technology identifies and records patient movement as they undergo medical examinations, receive treatments, or take rest periods, providing a real-time overview of patient activity on the premises. RTLS: Real-time location system.

**Figure S2 Overall research flow**


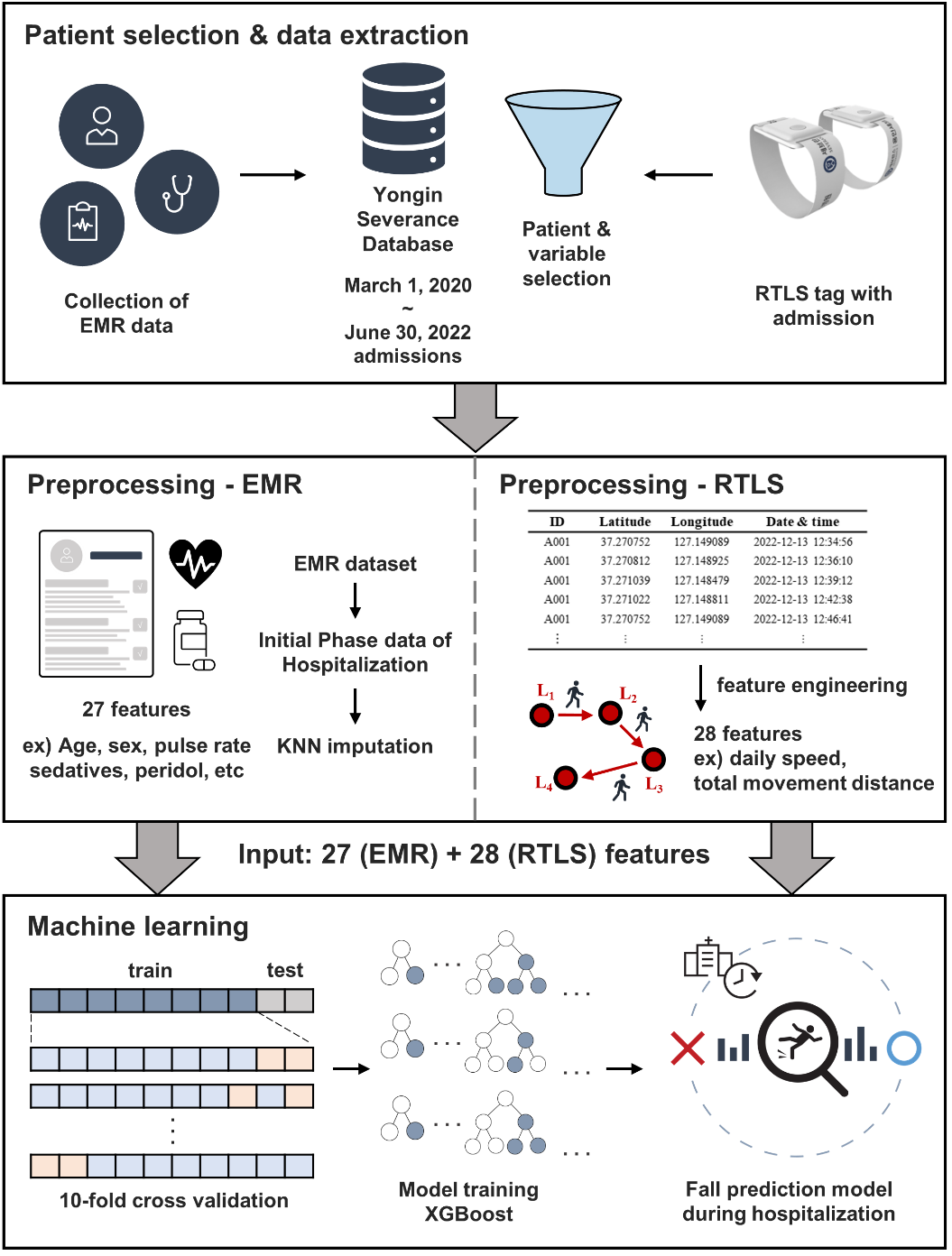


Figure S2 shows the framework of this study, which spanned from March 1, 2020 to June 30, 2022 and developed a predictive model for falls during hospital admissions using the EMR and RTLS data from the Yongin Severance Hospital. These data were obtained from patients who satisfied the inclusion criteria. The EMR data included 27 standard features procured during the initial phase of patient admission. The RTLS data, encompassing geographical coordinates and timestamps, were used to generate 28 additional features per patient, reflecting their physical activities. This comprehensive approach resulted in a dataset with 55 features, which served as the foundation for machine learning analysis. The Extreme Gradient Boost (XGBoost) algorithm was applied based on a 10-fold cross-validation method for fall prediction during hospital stay. EMR: electronic medical record; RTLS: real-time location system; KNN imputation: k-nearest neighbor imputation; XGBoost: the extreme gradient boost algorithm.

**Figure S3 Clinical variable distributions by fall status**
**A. Continuous variable density plots**


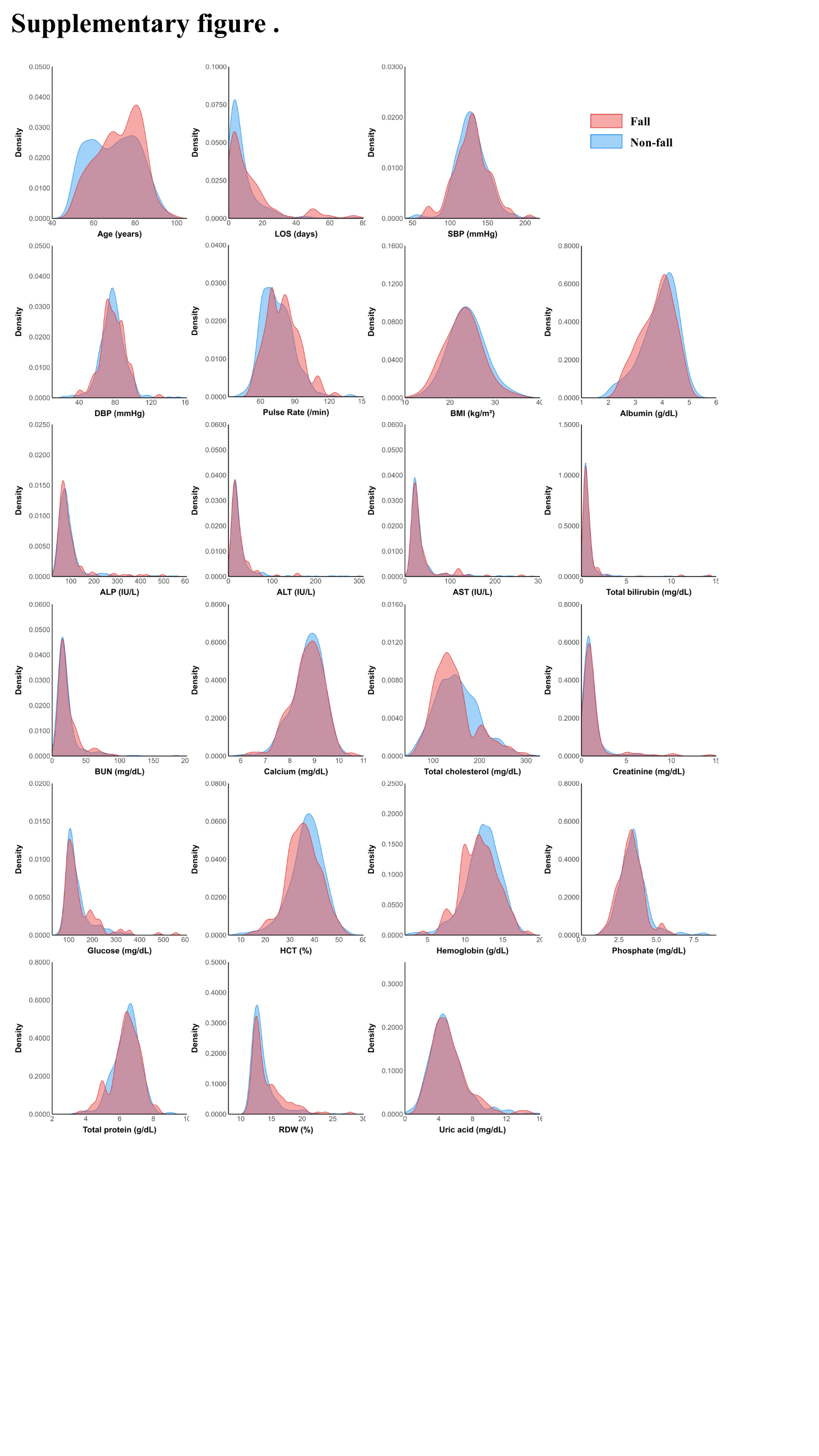


**B. Categorical variable proportions**


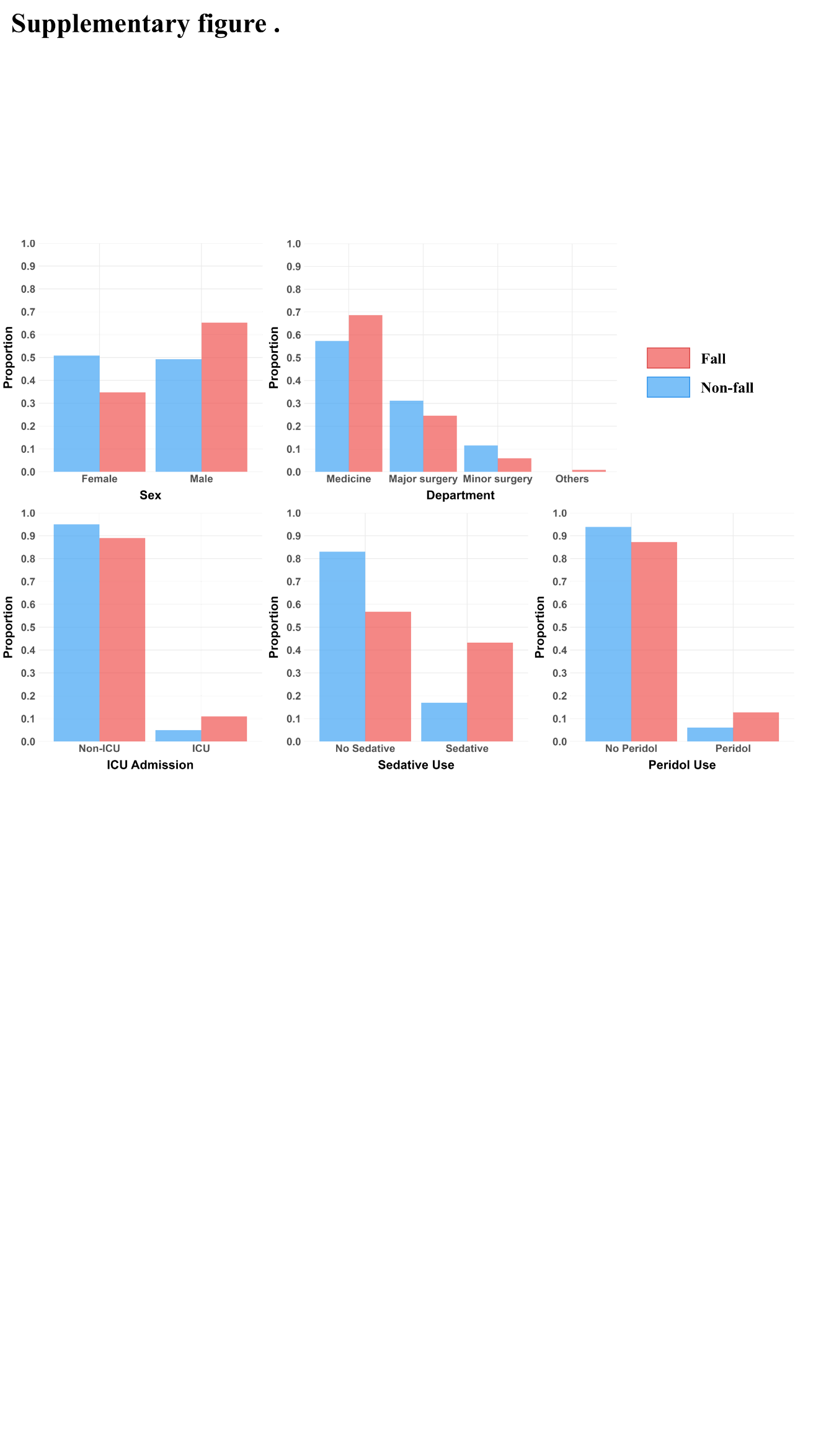


A, Continuous variable distributions, and B, categorical variable proportions between fall and non-fall groups. These plots highlight key clinical differences. Red represents fall patients, and blue represents non-fall patients. LOS: length of stay; SBP: systolic blood pressure; DBP: diastolic blood pressure; BMI: body mass index; ALP: alkaline phosphatase; ALT: alanine aminotransferase; AST: aspartate aminotransferase; BUN: blood urea nitrogen; HCT: hematocrit; RDW: red blood cell distribution width; ICU: intensive care unit.

**Figure S4 RTLS variable distributions by fall status**

**
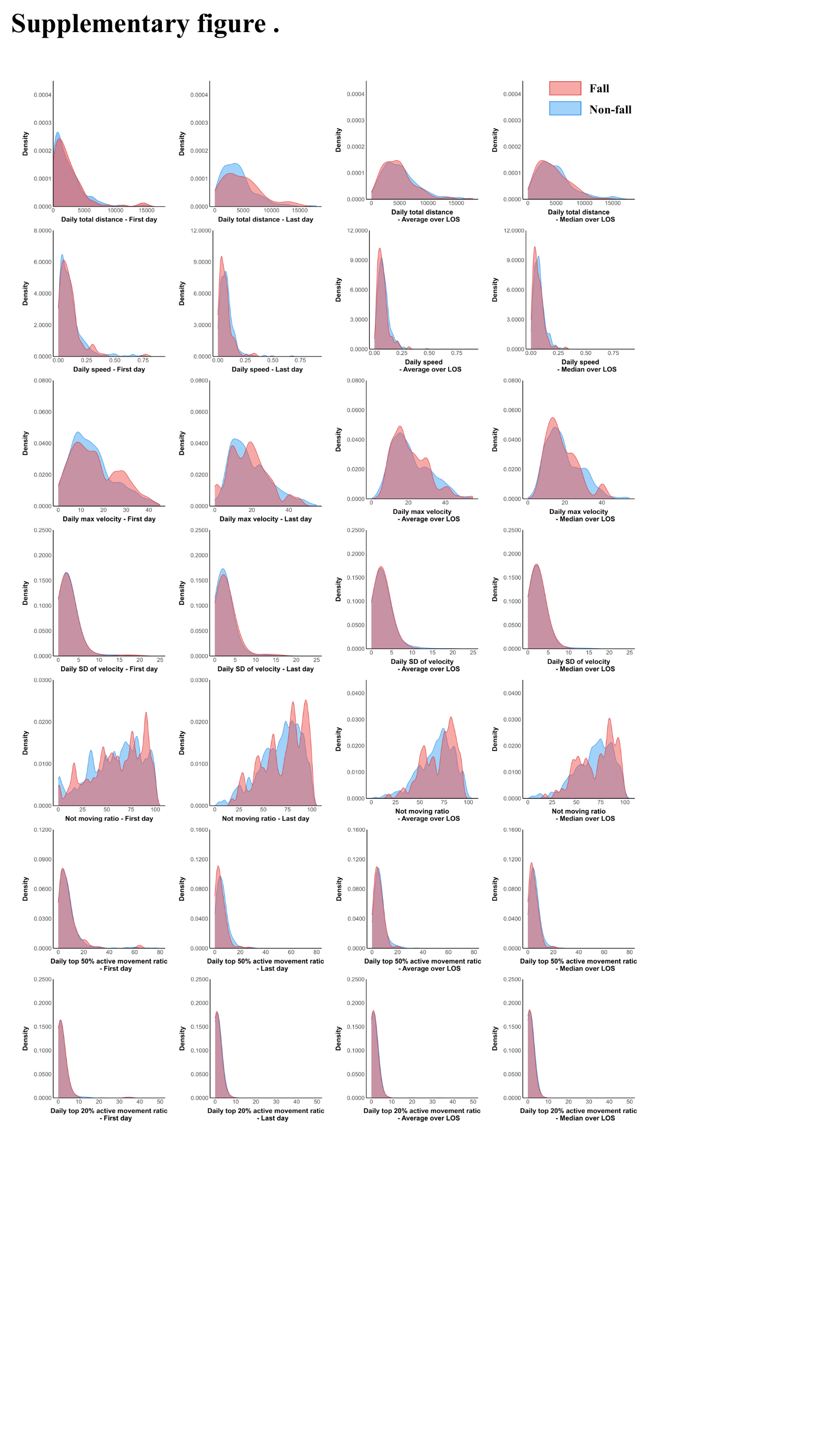
**
Density plots illustrate the distribution of RTLS variables for fall and non-fall groups. Red represents fall patients, and blue represents non-fall patients. LOS: length of stay; SD: standard deviation.

**Figure S5 Additional performance evaluation using the interval plots of the three models**

**
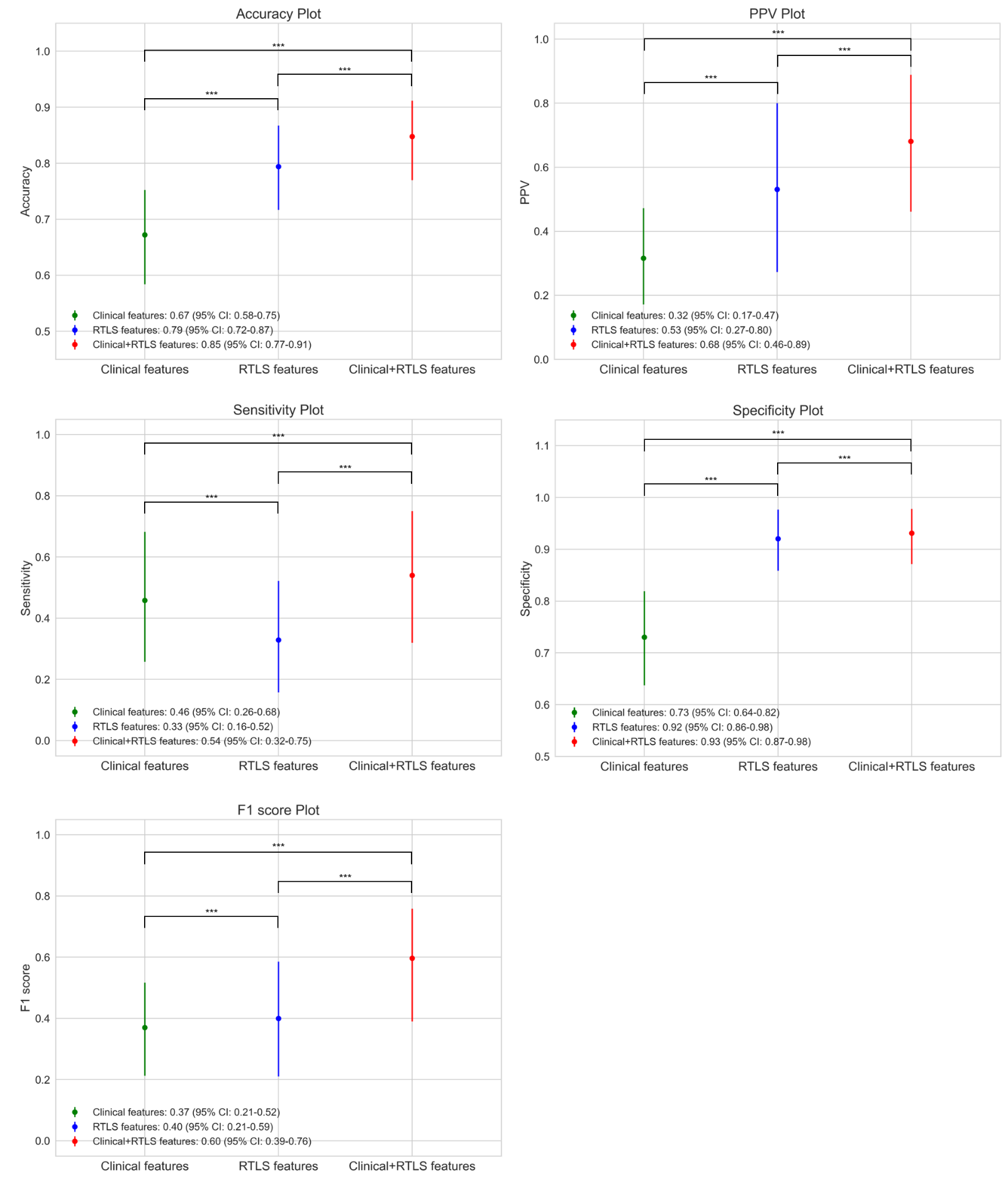
**

Performance comparison of the three models using accuracy, PPV, sensitivity, specificity, and F1 score based on 1000 bootstrap resamplings. The results are presented as 95% confidence intervals. Statistical significance is assessed using the Kruskal–Wallis test followed by Dunn’s post-hoc test, with significance levels indicated on the graph. The clinical, RTLS, and clinical + RTLS models are denoted in green, blue, and red, respectively. PPV: positive predictive value; CI: confidence interval.

**Figure S6 Feature importance of clinical and RTLS models using SHAP values**

**B. Feature importance of RTLS model**


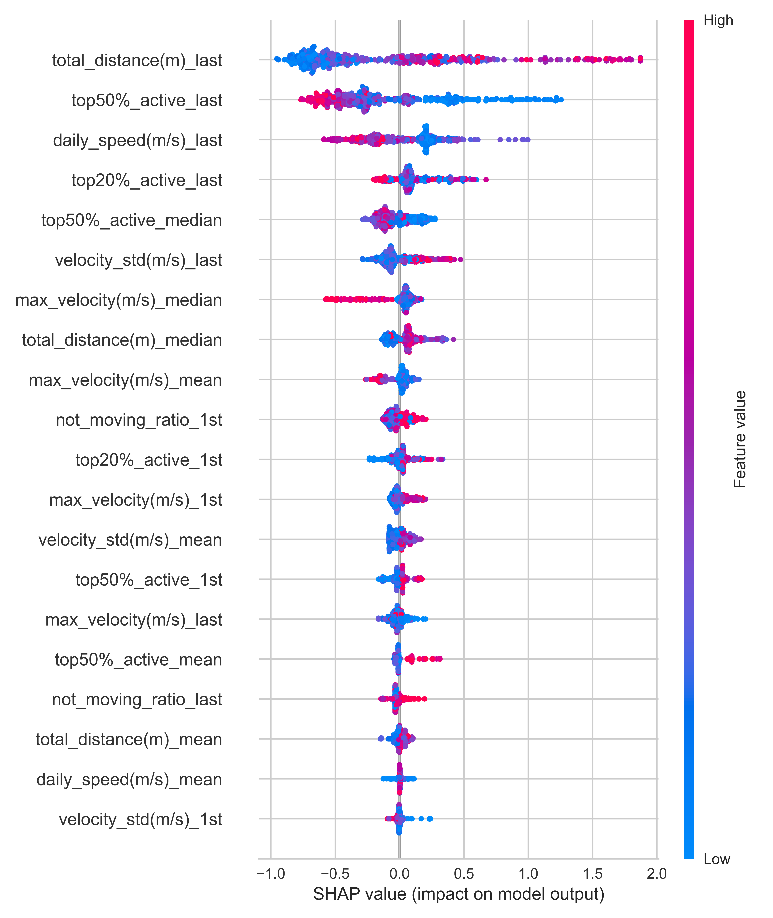


**A. Feature importance of clinical model**


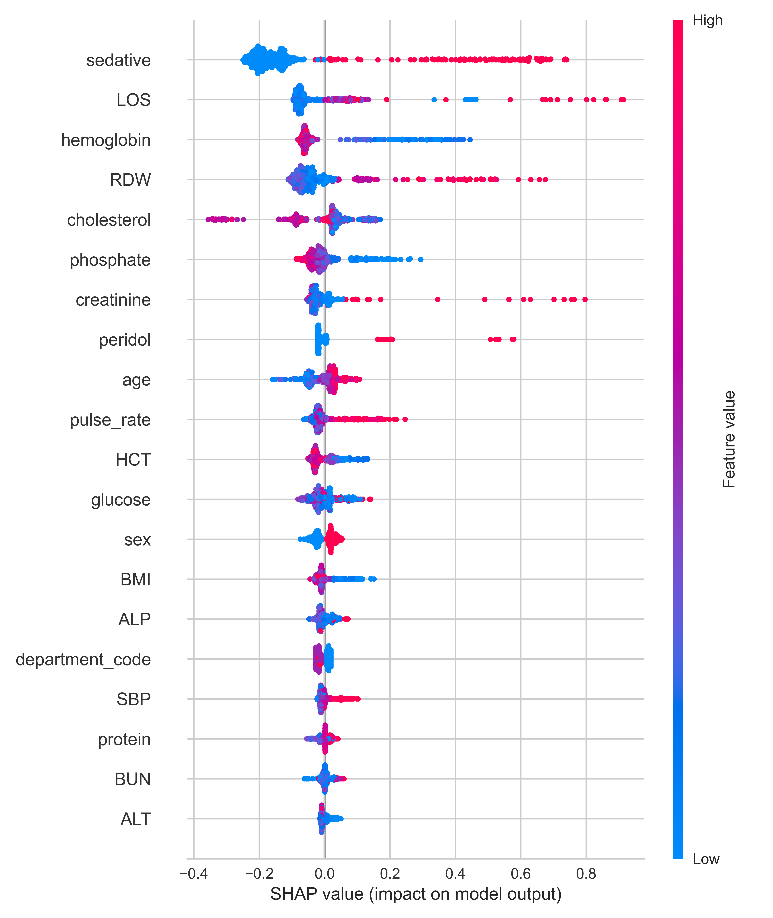

Impact of the top 20 features on model prediction ranked based on SHAP values. The y-axis displays the features in the order of decreasing impact, whereas the x-axis quantifies their SHAP values, with the color intensity ranging from blue for lower values to red for higher values. Specific descriptions of the clinical and RTLS features are listed in Tables S2 and S3, respectively. Top 20 important features of the A, clinical model and B, RTLS model determined via SHAP analysis. RTLS: real-time location system; SHAP value: Shapley additive explanation value; LOS: length of stay.

**Figure S7 Pearson correlation matrix of continuous variables**


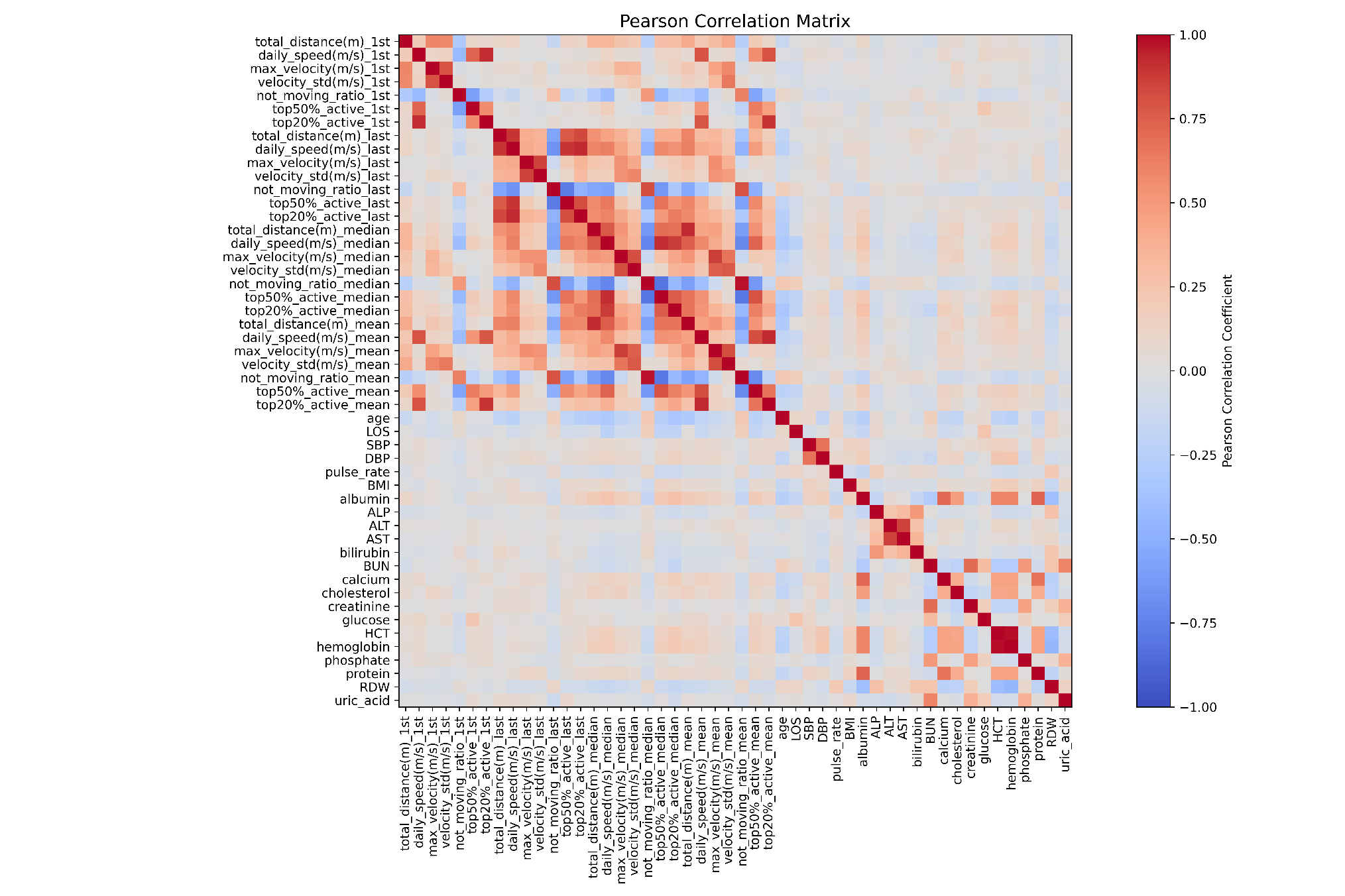


The Pearson correlation matrix displays the relationships between continuous variables in the clinical and RTLS models. Among the clinical and RTLS variables, the strongest correlations involving age were found with daily total distance (average over LOS) at -0.292, daily top 20% active movement ratio (median over LOS) at -0.330, and daily speed (median over LOS) at -0.310, all with p-values < 0.001. RTLS: real-time location system; LOS: length of stay; SBP: systolic blood pressure; DBP: diastolic blood pressure; ALP: serum alkaline phosphatase level; ALT: serum alanine aminotransferase level; AST: serum aspartate aminotransferase level; BUN: serum blood urea nitrogen level; HCT: whole blood hematocrit level; RDW: whole blood red cell distribution width.

**Figure S8** **SHAP Interaction Values Between Clinical and RTLS Features in the Clinical + RTLS Model**


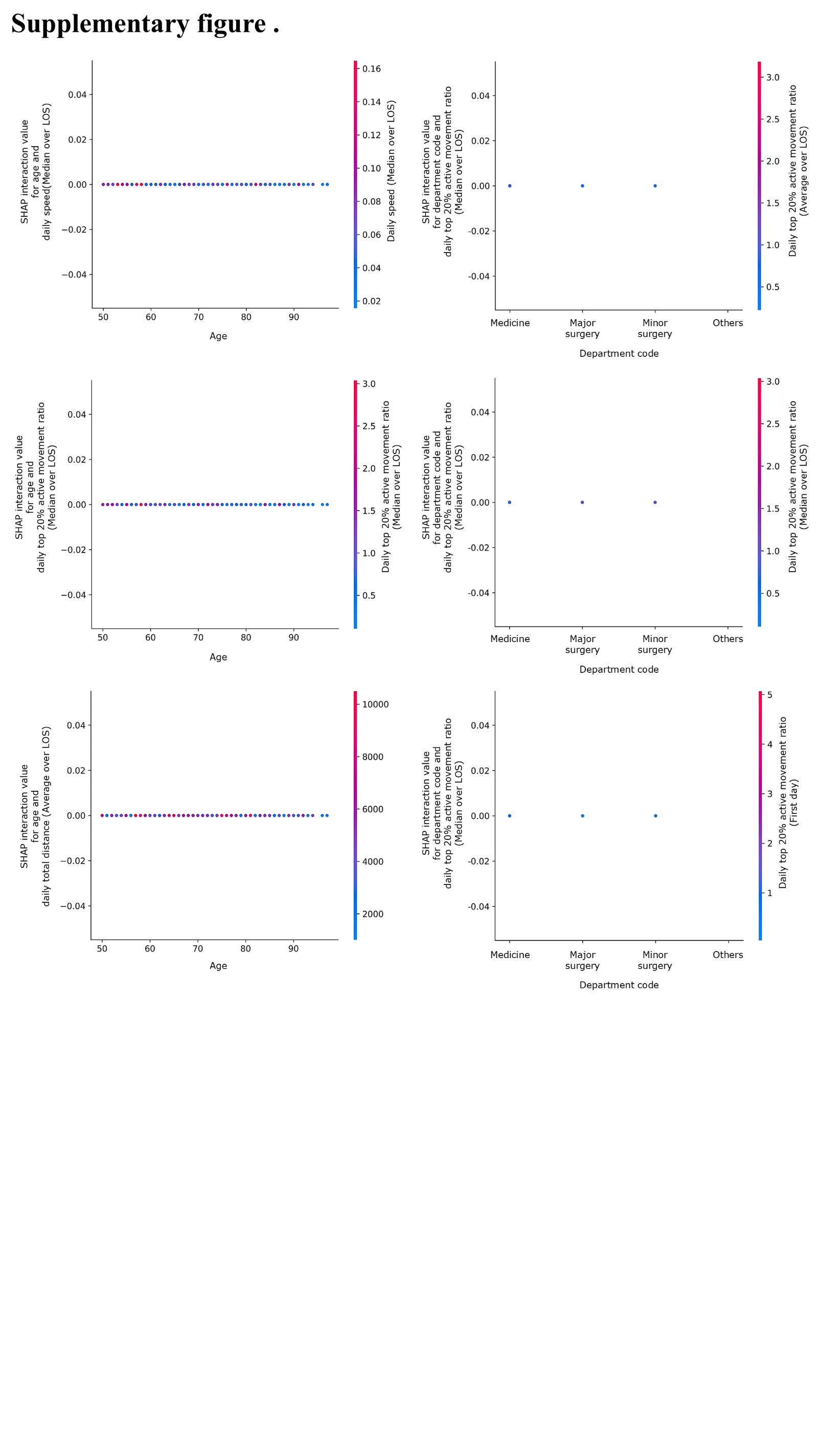


This figure illustrates SHAP interaction values for clinical and RTLS feature pairs with the highest correlations, as identified through Pearson’s correlation coefficient for continuous variables and Kruskal–Wallis test for categorical variables. The clinical and RTLS feature pairs presented are age with daily speed (median over LOS), age with daily top 20% active movement ratio (median over LOS), age with daily total distance (average over LOS), department code with daily top 20% active movement ratio (median over LOS), department code with daily top 20% active movement ratio (average over LOS), and department code with daily top 20% active movement ratio (first day). SHAP value: Shapley additive explanations value; RTLS: real-time location system; LOS: length of stay.

**Table S1 Department code classification**

| **Department code** | **Department** |
| --- | --- |
| Medicine | Family Medicine / Infectious Disease / Internal Medicine / Endocrinology / Rheumatology / Gastroenterology / Nephrology / Cardiology / Physical Medicine and Rehabilitation / Psychiatry / Hematology-Oncology / Pulmonology and Allergy / Respiratory Allergy |
| Major surgery | Hepato-Biliary-Pancreatic Surgery / Thyroid and Endocrine Surgery / Colorectal Surgery / Obstetrics and Gynecology / Neurosurgery / General Surgery / Gastrointestinal Surgery / Breast Surgery / Otolaryngology / Orthopedic Surgery / Thoracic Surgery |
| Minor surgery | Oral and Maxillofacial Surgery / Urology / Plastic Surgery /  Ophthalmology / Otorhinolaryngology / Dermatology |
| Others | Health Promotion Center / Anesthesiology and Pain Medicine / Emergency Medicine / Nuclear Medicine |

Classification of patient admissions into four department codes based on their department of hospitalization.

**Table S2** **Descriptions and counts of the missing clinical feature values**

| **Clinical variable** | **Feature name** | **Count of missing values** |
| --- | --- | --- |
| Sex | sex | 0 |
| Age | age | 0 |
| Length of stay | LOS | 0 |
| Department code | department_code | 0 |
| Intensive care unit (ICU) admission status | icu | 0 |
| Systolic blood pressure | SBP | 1 |
| Diastolic blood pressure | DBP | 1 |
| Pulse rate | pulse_rate | 1 |
| Body mass index (BMI) | BMI | 15 |
| Sedative intake status | sedative | 0 |
| Peridol intake status | peridol | 0 |
| Serum albumin level | albumin | 80 |
| Serum alkaline phosphatase (ALP) level | ALP | 85 |
| Serum alanine aminotransferase (ALT) level | ALT | 78 |
| Serum aspartate aminotransferase (AST) level | AST | 78 |
| Serum bilirubin level | bilirubin | 80 |
| Serum blood urea nitrogen (BUN) level | BUN | 73 |
| Serum calcium level | calcium | 82 |
| Serum total cholesterol level | cholesterol | 91 |
| Serum creatinine level | creatinine | 73 |
| Serum glucose level | glucose | 81 |
| Whole blood hematocrit level | HCT | 64 |
| Whole blood hemoglobin level | hemoglobin | 64 |
| Serum inorganic phosphate level | phosphate | 82 |
| Serum total protein level | protein | 80 |
| Whole blood red cell distribution width (RDW) | RDW | 64 |
| Serum uric acid level | uric_acid | 81 |

**Table S3 Description of RTLS features**

| **Mobility type** | **RTLS variable** | **Type** | **Feature name** | **Description** |
| --- | --- | --- | --- | --- |
| Amount of movement | Daily total distance (m) | First day | total_distance(m)_1st | Total distance moved (in meters) on the first day of RTLS records |
|  |  | Last day | total_distance(m)_last | Total distance moved (in meters) on the last day of RTLS records |
|  |  | Median | total_distance(m)_median | Median daily distance moved during hospitalization |
|  |  | Mean | total_distance(m)_mean | Average daily distance moved during hospitalization |
| Gait speed | Daily movement speed (m/s) | First day | daily_speed(m/s)_1st | Daily movement speed on the first day of RTLS records |
|  |  | Last day | daily_speed(m/s)_last | Daily movement speed on the last day of RTLS records |
|  |  | Median | daily_speed(m/s)_median | Median value of daily speed of movements during hospitalization |
|  |  | Mean | daily_speed(m/s)_mean | Mean value of daily speed of movements during hospitalization |
|  | Daily maximum movement velocity (m/s) | First day | max_velocity(m/s)_1st | Maximum velocity achieved on the first day of RTLS records |
|  |  | Last day | max_velocity(m/s)_last | Maximum velocity achieved on the last day of RTLS records |
|  |  | Median | max_velocity(m/s)_median | Median of daily maximum velocities recorded throughout hospitalization |
|  |  | Mean | max_velocity(m/s)_mean | Mean of daily maximum velocities recorded throughout hospitalization |
|  | Daily standard deviation  of velocity (m/s) | First day | velocity_std(m/s)_1st | Standard deviation of velocities on the first day of RTLS records |
|  |  | Last day | velocity_std(m/s)_last | Standard deviation of velocities on the last day of RTLS records |
|  |  | Median | velocity_std(m/s)_median | Median of daily velocity standard deviations throughout the hospitalization |
|  |  | Mean | velocity_std(m/s)_mean | Mean of daily velocity standard deviations throughout the hospitalization |
| Degree of active movement | Daily top 20% active movement ratio (%) | First day | top20%_active_1st | Ratio of time spent in the top 20% of active movements on the first day of RTLS records |
|  |  | Last day | top20%_active_last | Ratio of time spent in the top 20% of active movements on the last day of RTLS records |
|  |  | Median | top20%_active_median | Median of the daily ratio of time spent in the top 20% of active movements throughout the hospitalization |
|  |  | Mean | top20%_active_mean | Mean of the daily ratio of time spent in the top 20% of active movements throughout the hospitalization |
|  | Daily top 50% active movement ratio (%) | First day | top50%_active_1st | Ratio of time spent in the top 50% of active movements on the first day of RTLS records |
|  |  | Last day | top50%_active_last | Ratio of time spent in the top 50% of active movements on the last day of RTLS records |
|  |  | Median | top50%_active_median | Median of the daily ratio of time spent in the top 50% of active movements throughout the hospitalization |
|  |  | Mean | top50%_active_mean | Mean of the daily ratio of time spent in the top 50% of active movements throughout the hospitalization |
|  | Daily not moving ratio (%) | First day | not_moving_ratio_1st | Ratio of time without movement  on the first day of RTLS records |
|  |  | Last day | not_moving_ratio_last | Ratio of time without movement  on the last day of RTLS records |
|  |  | Median | not_moving_ratio_median | Median of the daily ratios of time  without movement throughout the hospitalization |
|  |  | Mean | not_moving_ratio_mean | Mean of the daily ratios of time  without movement throughout the hospitalization |

RTLS: real-time location system.

**Table S4** **Baseline characteristics (RTLS features) of patients**

|  | **Overall (n=561)** | **Fall (n=118)** | **No-fall (n=443)** | **P-value** |
| --- | --- | --- | --- | --- |
| **Daily total distance (m)** |  |  |  |  |
| **First day** | 1719.9 [700.7-3405.2] | 1773.0 [778.0-3261.9] | 1718·8 [685.8-3434.6] | 0.753 |
| **Last day** | 3710.8 [2049.5-5685.4] | 4509.5 [2351.7-6675.1] | 3640·8 [1990.3-5427.6] | 0.055 |
| **Average over LOS** | 4610.1 [2821.7-6530.0] | 4410.2 [2586.5-5979.2] | 4683·6 [2832.7-6640.5] | 0.225 |
| **Median over LOS** | 4173.3 [2408.9-6166.0] | 4055.2 [2196.2-6077.6] | 4228·0 [2484.1-6221.5] | 0.363 |
| **Daily speed (m/s)** |  |  |  |  |
| **First day** | 0.08 [0.04-0.13] | 0.08 [0.04-0.13] | 0.08 [0.04-0.14] | 0.819 |
| **Last day** | 0.07 [0.04-0.10] | 0.06 [0.03-0.09] | 0.07 [0.04-0.10] | 0.008 |
| **Average over LOS** | 0.07 [0.05-0.11] | 0.06 [0.04-0.09] | 0.08 [0.05-0.11] | 0.023 |
| **Median over LOS** | 0.07 [0.04-0.10] | 0.05 [0.03-0.09] | 0.07 [0.04-0.10] | 0.019 |
| **Daily max velocity (m/s)** |  |  |  |  |
| **First day** | 14.1 [8.2-23.5] | 15.0 [8.3-25.3] | 14.0 [8.2-21.9] | 0.582 |
| **Last day** | 18.0 [11.0-28.0] | 19.0 [10.9-27.8] | 17.5 [11.1-28.0] | 0.751 |
| **Average over LOS** | 20.3 [14.2-31.2] | 19.9 [14.5-29.3] | 20.4 [14.2-32.3] | 0.441 |
| **Median over LOS** | 17.9 [12.6-26.1] | 16.7 [12.5-24.2] | 18.1 [12.6-26.8] | 0.268 |
| **Daily SD of velocity** |  |  |  |  |
| **First day** | 2.7 (4.9) | 2.5 (2.5) | 2.7 (5.3) | 0.512 |
| **Last day** | 2.0 [1.4-2.8] | 2.1 [1.4-3.1] | 2.0 [1.4-2.6] | 0.2 |
| **Average over LOS** | 2.2 [1.7-3.2] | 2.3 [1.6-3.2] | 2.2 [1.7-3.2] | 0.854 |
| **Median over LOS** | 2.0 [1.6-2.8] | 2.0 [1.5-2.8] | 2.0 [1.6-2.9] | 0.676 |
| **Daily not moving ratio** |  |  |  |  |
| **First day** | 63.1 [40.0-80.7] | 63.0 [43.1-83.9] | 63.2 [38.9-80.3] | 0.573 |
| **Last day** | 72.1 [54.1-84.7] | 76.8 [55.9-89.0] | 71.2 [53.4-83.8] | 0.085 |
| **Average over LOS** | 70.8 [56.2-81.2] | 74.3 [56.1-82.8] | 69.7 [56.4-80.0] | 0.145 |
| **Median over LOS** | 72.2 [56.1-84.0] | 75.8 [55.6-86.5] | 71.2 [56.2-83.2] | 0.14 |
| **Daily top 50% active movement ratio** |  |  |  |  |
| **First day** | 5.8 [2.5-9.8] | 5.6 [2.5-9.9] | 5.8 [2.6-9.7] | 0.874 |
| **Last day** | 4.8 [2.4-7.5] | 3.4 [1.7-6.9] | 5.2 [2.7-7.8] | 0.001 |
| **Average over LOS** | 5.1 [3.0-7.6] | 4.6 [2.7-7.0] | 5.2 [3.3-7.7] | 0.081 |
| **Median over LOS** | 4.6 [2.5-7.2] | 3.5 [2.0-6.4] | 4.8 [2.7-7.3] | 0.012 |
| **Daily top 20% active movement ratio** |  |  |  |  |
| **First day** | 1.0 [0.3-2.2] | 1.1 [0.3-2.2] | 1.0 [0.3-2.2] | 0.967 |
| **Last day** | 0.9 [0.4-1.6] | 0.8 [0.4-1.3] | 0.9 [0.4-1.7] | 0.035 |
| **Average over LOS** | 1.0 [0.6-1.6] | 0.9 [0.5-1.4] | 1.1 [0.6-1.7] | 0.03 |
| **Median over LOS** | 0.9 [0.4-1.5] | 0.7 [0.4-1.2] | 1.0 [0.5-1.6] | 0.01 |

Data are presented as median (IQR) and mean (SD) for non-normally and normally distributed data, respectively. Units are specified next to each variable. The Shapiro–Wilk test was used to evaluate the normality of continuous variables. The t-test was employed for data satisfying the normality criteria, whereas the Mann–Whitney U test was applied for data not adhering to a normal distribution. LOS: length of stay; SD: standard deviation.

**Table S5 Additional comparative performance metrics of the three models**

| **Model** | **Youden index** | **Accuracy** | **PPV** | **Sensitivity** | **Specificity** | **F1 score** |
| --- | --- | --- | --- | --- | --- | --- |
| Clinical model | 0.298 | 0.672 (0.584-0.752) | 0.316 (0.171-0.472) | 0.458 (0.258-0.682) | 0.730 (0.637-0.819) | 0.370 (0.213-0.517) |
| RTLS model | 0.428 | 0.794 (0.717-0.867) | 0.581 (0.272-0.800) | 0.329 (0.158-0.522) | 0.921 (0.859-0.977) | 0.400 (0.211-0.585) |
| Clinical + RTLS model | 0.376 | 0.848 (0.770-0.912) | 0.681 (0.462-0.889) | 0.540 (0.320-0.750) | 0.931 (0.872-0.978) | 0.600 (0.390-0.759) |

The displayed values are the performance metrics for the clinical, RTLS, and clinical + RTLS models, with 95% confidence intervals (CIs) in parentheses. RTLS: real-time location system; PPV: positive predictive value.

**Table S6** **Subgroup analysis of model performance based on specific clinical conditions**

|  |  |  | **Clinical model** | | **RTLS model** | | **Clinical + RTLS model** | |
| --- | --- | --- | --- | --- | --- | --- | --- | --- |
| **Subgroup** | **Category** | **n** | **AUROC** | **P-value** | **AUROC** | **P-value** | **AUROC** | **P-value** |
| **Age** | **≤ 65 years** | 44 | 0.794 | 0.164 | 0.778 | 0.676 | 0.895 | 0.489 |
|  | **> 65 years** | 69 | 0.644 |  | 0.827 |  | 0.841 |  |
| **Sex** | **Female** | 48 | 0.666 | 0.637 | 0.822 | 0.890 | 0.894 | 0.382 |
|  | **Male** | 65 | 0.719 |  | 0.807 |  | 0.825 |  |
| **Department code** | **Medicine** | 74 | 0.725 | 0.973 | 0.769 | 0.238 | 0.855 | 0.742 |
|  | **Surgery** | 38 | 0.729 |  | 0.879 |  | 0.825 |  |
| **LOS** | **≤ 7 days** | 70 | 0.682 | 0.996 | 0.745 | 0.280 | 0.855 | 0.775 |
|  | **> 7 days** | 43 | 0.682 |  | 0.867 |  | 0.830 |  |

Displayed values represent the AUROC for the clinical, RTLS, and clinical + RTLS models across different subgroups, including age, sex, department, and LOS. P-values were calculated using DeLong’s test to assess the significance of differences between subgroups. RTLS: real-time location system; AUROC: area under the receiver operating characteristic curve; LOS: length of stay.
